# Biconnectivity of the cellular metabolism: A cross-species study and its implication for human diseases

**DOI:** 10.1038/srep15567

**Published:** 2015-10-22

**Authors:** P. Kim, D.-S. Lee, B. Kahng

**Affiliations:** 1 Center for Complex Systems Studies and CTP, Department of Physics and Astronomy, Seoul National University, Seoul 151-747, Korea; 2 Department of Physics, Inha University, Incheon 402-751, Korea

## Abstract

The maintenance of stability during perturbations is essential for living organisms, and cellular networks organize multiple pathways to enable elements to remain connected and communicate, even when some pathways are broken. Here, we evaluated the biconnectivity of the metabolic networks of 506 species in terms of the clustering coefficients and the largest biconnected components (LBCs), wherein a biconnected component (BC) indicates a set of nodes in which every pair is connected by more than one path. Via comparison with the rewired networks, we illustrated how biconnectivity in cellular metabolism is achieved on small and large scales. Defining the biconnectivity of individual metabolic compounds by counting the number of species in which the compound belonged to the LBC, we demonstrated that biconnectivity is significantly correlated with the evolutionary age and functional importance of a compound. The prevalence of diseases associated with each metabolic compound quantifies the compounds vulnerability, i.e., the likelihood that it will cause a metabolic disorder. Moreover, the vulnerability depends on both the biconnectivity and the lethality of the compound. This fact can be used in drug discovery and medical treatments.

Cellular metabolism enables the execution of numerous cellular functions on the basis of the genetic information contained within living organisms. Metabolic reactions and compounds are intricately wired, and their organization may change during evolution in fluctuating environments over long periods of time^1^,^2^,^3^,^4^,^5^. The structures of various cellular networks have been studied for the past decade^6^,^7^,^8^,^9^,^10^,^11^,^12^,^13^,^14^,^15^ and are quite different from the structures of random networks^16^,^17^,^18^; they are characterized by enhanced modularity^19^,^20^ and a power-law distribution of the number of links (degree) of each node^21^,^22^. Efforts have been made to understand the origin and implications of the anomalous yet universal features of biological networks. For example, the gene duplications and mutations that accompany the inheritance of interaction links have been proposed as the mechanisms that result in broad degree distributions in cellular networks^5^. The topology of human cellular networks has also proven to be relevant to the pathogenesis and prevalence of clinical disorders^23^,^24^,^25^,^26^,^27^.

In a networked system, a path is necessary for the communication and interaction of two elements. A connected component, which is a set of nodes wherein every pair of nodes is connected by one or more paths, can act as a functional module, which comprises smaller modules in charge of different roles. The largest connected component (LCC) contains the core part of a given network^28^. Theoretical studies have demonstrated that networks with power-law degree distributions, referred to as scale-free (SF) networks, are capable of forming an LCC that is comparable in size to the total number of nodes, even with only a small number of links. This finding suggests that SF networks can reliably execute their core functions, even with the loss of a significant fraction of links^29^.

Small perturbations are often more common than large ones. Cellular networks need to maintain their regularity during small but frequent perturbations; therefore, a single path between two elements may be insufficient for the robust maintenance of their normal states. If more than one disjoint path exists between two nodes, the nodes can remain connected and therefore function normally, even with some loss in one path. The impact of multiple pathways has recently been investigated in various contexts, including the epistatic interactions of different enzymes in yeast metabolism^30^, the pair-wise interactions of drugs^31^, and the synthetic lethality of bacterial metabolism^32^,^33^.

The reaction pathways of metabolism are organized for the survival and reproduction of a species^34^. If there are alternatives for many pathways, the robustness under perturbation is increased, thus endowing a species with a high degree of resilience. However, the establishment of backup pathways may have a biochemical and entropic cost; the establishment of a pathway can require biochemical resources, and it can take substantial time for a mutant equipped with a backup pathway of interest to appear. Therefore, the organization of alternative or backup pathways can indicate the ways in which living organisms remodel their cellular networks towards high resilience during evolution. In this work, we analyzed the structures of the metabolic networks of hundreds of living species to determine the organization of backup pathways. We consulted the BioCyc database version 13.1^8^ to construct the bipartite metabolic networks of 506 species and to numerically determine their clustering coefficients and biconnected components (BCs). The clustering coefficient quantifies the abundance of diamond-shaped subgraphs that involve two distinct reactions that process two metabolites. In graph theory, two nodes are considered to be biconnected if there are two disjoint paths connecting them. A BC is a subset of nodes and links in which every pair of nodes is connected by two or more disjoint paths and therefore is expected to be robust against perturbations because most pairs of nodes would remain connected even if some pathways break.

We demonstrated that real networks have, compared with randomly wired networks that preserve degree distributions of both metabolites and reactions, greater clustering coefficient but smaller largest biconnected component(LBC), and indicating the scale-dependent organization of pathways for the maintenance of order. This feature is preserved even after coarse graining of nodes into modules. We subsequently defined the biconnectivity of individual compounds by counting the number of species in which the compound belonged to the LBCs. The establishment of a detour pathway is determined by the competition in terms of cost and benefit. Evolutionarily old compounds, identified here with those present in many species, were demonstrated to have high biconnectivity because they are associated with many backup pathways. Additionally, the important compounds, which may be lethal if their concentrations reach abnormal levels and which were identified with compounds located close to the biomass components, exhibited high biconnectivity, thus suggesting that abundant backup pathways are associated with compounds of functional importance. The biconnectivity of each compound is related to the maintainability of its normal state. Therefore, the likelihood of disease occurrence related to a compound is expected to depend on the compounds biconnectivity. By identifying human diseases, the pathogenesis of which could potentially involve the abnormality of each metabolic compound^8^,^24^,^25^,^35^,^36^,^37^, we demonstrated that the prevalence of the associated diseases was negatively correlated with biconnectivity. Disease prevalence should also depend on the lethality of the compound, and we demonstrated that the correlation between the biconnectivity and the lethality of metabolic compounds facilitates an understanding of the disease prevalence patterns.

The work we present here is an extensive and quantitative analysis of pathway organization across hundreds of species. The obtained results offer insights into the evolutionary pressure imposed on the topology of metabolic networks that should be stable while interacting ceaselessly with fluctuating environments. We discuss the implications of our findings for human diseases and drug target searches.

## Results

### Biconnectivity of the metabolic network of each species

**Figure 1:**
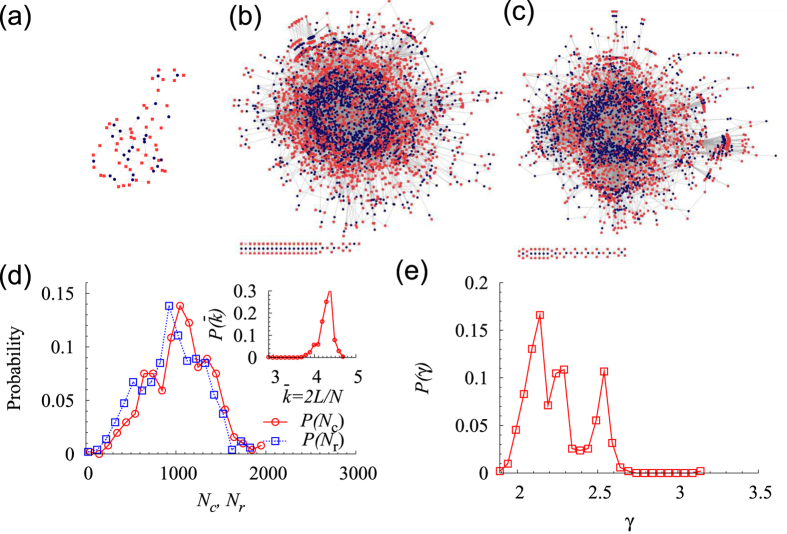
Properties of the metabolic networks of the 506 species investigated. (**a**–**c**) Bipartite networks of compounds and reactions of (**a**) *Ashbya gossypii ATCC 10895* (*N*_*c*_ = 46 and *N*_*r*_ = 24), which is known to have the smallest genome among eukaryotes^60^. (**b**) *Burkholderia sp.* (*N*_*c*_ = 1951 and *N*_*r*_ = 1888) and (**c**) human (*N*_*c*_ = 1513 and *N*_*r*_ = 1451). Here, *N*_*c*_ and *N*_*r*_ represent the numbers of compounds (red squares) and reactions (blue circles). Among the 506 species considered in this work, *Burkholderia sp.* has the largest metabolism and *Ashbya gossypii* has the smallest. (**d**) Distribution of *N*_*c*_ and *N*_*r*_. Inset: Distribution of the mean degree 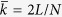 with 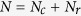. (**e**) Distribution of the exponent *γ*, which characterizes the degree distribution for compounds 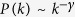.

In the bipartite metabolic network for each species, a reaction is connected by undirected links to all its substrates and products [Fig. 1(a–c)]. Although the reversibility of individual reactions may provide important information, some irreversible reactions may be forced in the reverse direction under certain physiological conditions of temperature or metabolite concentrations^38^,^39^,^40^. The numbers of metabolic reactions, *N*_*r*_, compounds, *N*_*c*_, and links, *L*, are not broadly distributed over species [Fig. 1(d)]. In contrast, the number of reactions that process a compound is highly heterogeneous. The distribution of the degree *k* of a compound follows a power-law 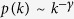, with the exponent *γ*, which ranges between 2 and 3 [Fig. 1(e)]^6^.

In this study, we applied the compound-centric view in considering the biconnectivity of the metabolic networks, because the stable production and consumption of metabolites is the most important function of metabolism. Moreover, the presence of two distinct paths is more meaningful for a pair of compounds than for a pair of reactions. When two metabolites are connected by two pathways, the production of one metabolite from the other is conducted along two pathways and can occur even if one pathway is broken. However, two pathways that connect two reactions do not guarantee the stability of both reactions.

**Figure 2:**
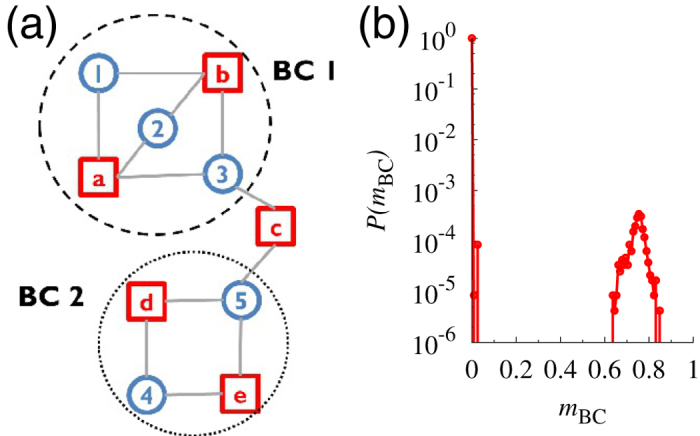
Biconnected components (BC). (**a**) A network of 5 compound nodes (red squares) and 5 reaction nodes (blue circles) has two BCs. BC 1, of size 5, is the LBC; thus, *m*_2_ of this network is 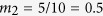. (**b**) Distribution of the relative size 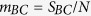 of a BC in the 506 species considered. Note that the fraction of nodes in each BC other than the LBC is less than 0.05.

The clustering coefficient of each metabolic network was computed, as described in Methods. Using the algorithm in ref. 41, we obtained the connected components and the BCs of each metabolic network [Fig. 2(a)]. A metabolic network typically consists of a single LCC and many small connected components. The LBC lies inside the LCC, and the fraction of the nodes that belong to the LBC, 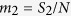, with *S*_2_ the number of nodes in the LBC and 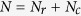, characterizes the biconnectivity of a network on the largest scale. Except for the LBC, other BCs are quite small in a given metabolic network [Fig. 2(b)].

For comparison, we generated three ensembles of rewired networks; they are obtained by relocating of randomly selected links completely randomly (‘rewired-all’), by changing the end reactions of the links to randomly selected reactions, which preserves the degree of every compound (‘rewired-c’), and by exchanging the neighbor reactions of two metabolites via crosslinking, which preserves the degree distributions of both compounds and reactions (‘rewired-cr’).

**Figure 3:**
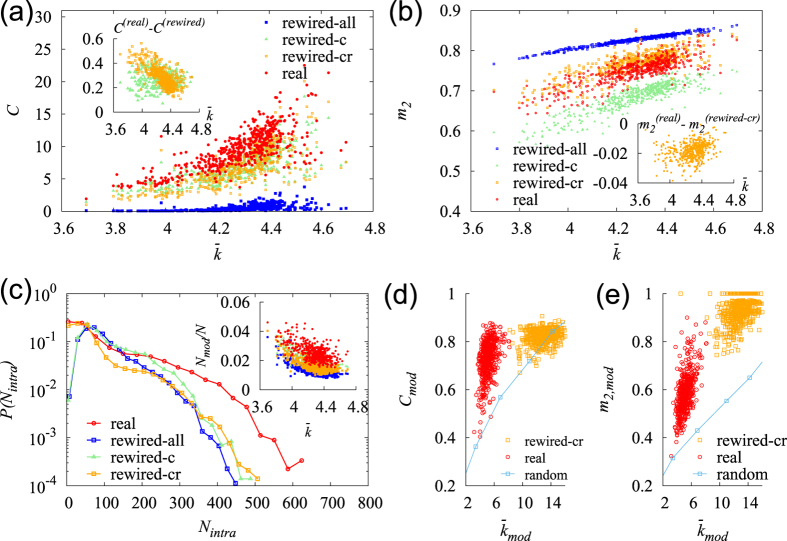
Biconnectivity of the metabolic networks. (**a**) Plot of the clustering coefficient *C* versus the mean degree 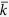. Each of the 506 data points grouped as ‘rewired-all’ (‘rewired-c’, ‘rewired-cr’) is the average over 100 rewired-all (rewired-c, rewired-cr) networks generated for each species. Inset: Both 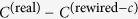 and 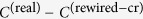 are positive, which indicates that *C*^(real)^ is larger than the rewired networks. (**b**) Plot of the fraction of the LBC, *m*_2_ versus the mean degree 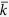. Inset: 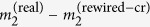 is negative, which indicates that the *m*_2_ of the real networks is smaller than the rewired-cr networks. (**c**) Distribution of the size of a module *N*_intra_. *P*(*N*_intra_) is broader for the real networks than the rewired networks. Inset: Plot of the number of modules *N*_mod_ versus 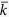. (**d**) Plot of the clustering coefficient *C*_mod_ versus the mean degree 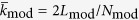 for the modular networks. The modular network is a unipartite network; thus, *C*_mod_ is evaluated following the conventional definition, 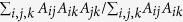 where *A*_*ij*_ is the adjacency matrix of the modular network. (**e**) Plot of *m*_2,*mod*_ versus the mean degree 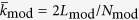 for the modular networks.

The clustering coefficients, *C*, and the fractions of the LBC, *m*_2_, were plotted as a function of the mean degree 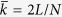 for the real and the rewired networks of the 506 species in Fig. 3(a,b). On average, 75% of the nodes of the real networks belonged to the LBC, i.e., 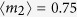 and 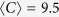. The clustering coefficient was greater than any of the considered rewired networks, thus suggesting enhanced biconnectivity on a small scale. The fraction of the LBC was smaller than the rewired-all and the rewired-cr networks; however, it was larger than the rewired-c networks.

It has been shown^42^,^43^ that *m*_2_ depends on 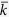 and the large-*k* behavior of the degree distribution *p*(*k*). When 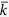 is not too small, the random SF networks have smaller LBCs than the Erdös-Rény (ER) networks, which are constructed by randomly assigning links and thus have narrow Poisson degree distributions. Therefore, the order of the *m*_2_s of the real, rewired-all, and rewired-c networks can be understood by noting that the rewired-all networks have narrower degree distributions for both reactions and compounds compared with the real networks, whereas the rewired-c networks have broader degree distributions for reactions than the real networks. It should be noted, however, that in large-scale failures in which a significant number of links are lost and 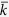 becomes quite small, hub nodes, the abundance of which is responsible for broad degree distributions, play a dominant role as seeds for building connected components and BCs, leading to larger LCCs and LBCs than without hubs^43^,^44^. Therefore, the abundance of hub nodes facilitates large-scale failure survival.

The topology of metabolic networks exhibits various features, such as modularity^19^,^20^, which may be responsible for the difference between the real networks and the rewired-cr networks. Cellular metabolism consists of biochemical pathways, such as glycolysis, the TCA cycle, and fatty acid pathways^45^, and the graph-theoretical approaches enable us to computationally identify the modules that exhibit high intraconnectivity and low interconnectivity^20^,^46^. In the application of the algorithm utilizing the maximization of modularity^47^, one can obtain the modules, which provide a coarse-grained view of the cellular metabolism in each species. We identified a larger number of modules *N*_mod_ in the real networks than any ensemble of the rewired networks. These modules were non-uniform in size compared with the rewired networks [Fig. 3(c)]. The networks of *N*_mod_ nodes and *L*_mod_ links, in which two nodes (modules) are connected if a link connects the nodes that belong to them in the original network, exhibit clustering coefficients and LBCs larger than the modular networks extracted from the ER networks [blue lines in Fig. 3(d,e)] for a given mean degree 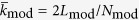. In the real networks, compared with the modular networks from the rewired-cr networks, the modular clustering coefficients were similar; however, the modular LBC, *m*_2,*mod*_, was substantially larger in the rewired-cr networks than the real networks, similar to *m*_2_. Given the prominent modularity of metabolic networks^19^,^20^, the smaller values of *m*_2_, a measure of the long-range biconnectivity, of the real networks than the rewired-cr networks can be related to the larger number of modules and their low biconnectivity. The backup pathways are subsequently expected to be selectively established for pathways that require a high degree of stability.

Although the specific modules in a given network may differ depending on the algorithm used, we demonstrated that our results were not changed even when we used a different algorithm, such as the algorithm in^48^ (Fig. S2). To further confirm the robustness of our results, we performed the same analysis after removal of the highly abundant compounds that are involved in numerous reactions, such as protons, water, adenosine triphosphate (ATP), and carbon dioxide^49^, in the metabolic networks of the studied species, and our results remained valid (Fig. S3). For example, even without currency metabolites^49^, the fraction of the LBC is only mildly changed 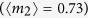. We also analyzed the well-curated metabolic reconstructions of two strains of *Escherichia coli* and *Helicobacter pylori, Pseudomonas putida, Staphylococcus aureus, Methanosarcina barkeri, Mycobacterium tuberculosis, Saccharomyces cerevisiae*, and *Homo sapiens* from the BiGG database^50^; our results did not qualitatively change, although some trends were difficult to identify because of the small number of data points [Fig. S4].

The evolution of species, including speciation, may have occurred along with the recruitment of new pathways, which enhanced stability, or the removal of those that were merely redundant, thus leading to the gradual variation of the biconnectivity of metabolism^1^,^2^. Using the National Center for Biotechnology Information (NCBI) taxonomy database for species classification according to morphological studies and molecular information^51^,^52^, we computed the network distance, *d*_*ij*_, which represents the length of the shortest path in the phylogenetic tree, between every pair of species *i* and *j* to define as the evolutionary distance. Considering the normalized difference of *m*_2_ between two species *i* and *j*, defined as 
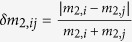
, we demonstrated that 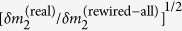 increased with the evolutionary distance *d*_*ij*_; the Pearson correlation coefficient (*PCC*) was 0.017, and the P-value (*P*) was smaller than 10^−8^. The exponent 1/2 was introduced to allow the small values of 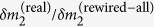 to significantly contribute to the average over species pairs.

### Biconnectivity of individual metabolic compounds

The cross-species analysis of the BCs of the metabolic networks indicates the heterogeneity of the metabolic compounds that participate in cellular metabolism. Let us equate the ratio of the number of species that have a compound *i* in metabolism to the total number of all considered species by the popularity *f*_*i*_^53^. Compounds with a large *f*, and the enzymes that bind to them, may have been introduced to metabolism in the early stages of evolution and inherited by many contemporary species. Therefore, compound popularity can be a measure of evolutionary age. The popularity is broadly distributed, and follows a power-law distribution: 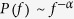, where 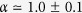.

**Figure 4:**
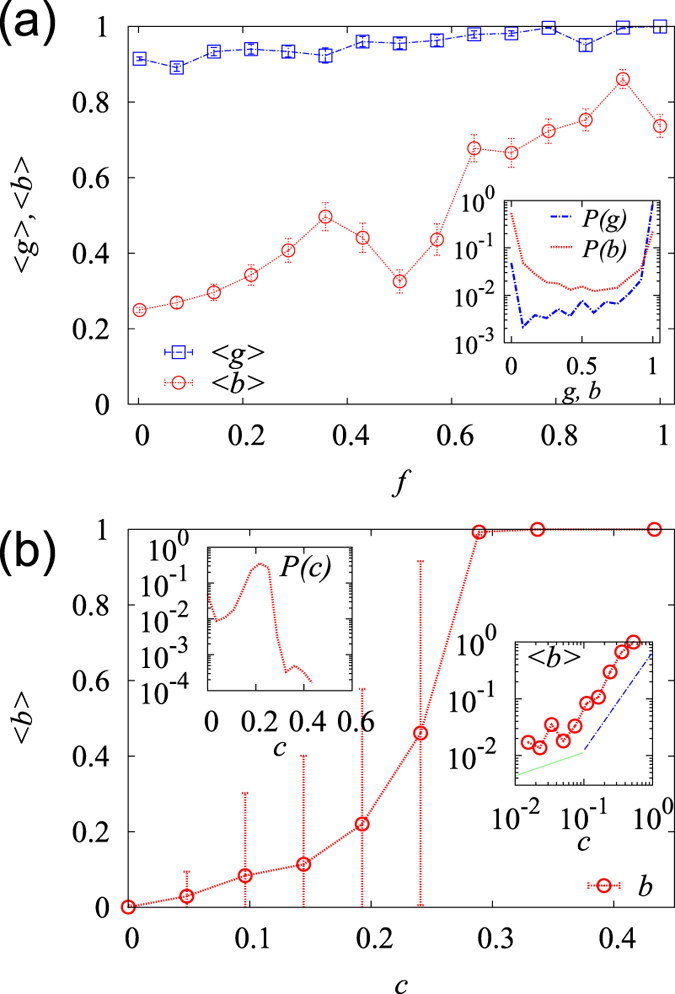
The cross-species biconnectivity of the metabolic compounds. (**a**) The average (single-) connectivity 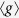 and the biconnectivity 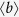 of the compounds of given popularity *f*. The correlation between *g* and *f* was *PCC* = 0.102 and between *b* and *f* was *PCC* = 0.358. Inset: The distributions of *g* and *b*. (**b**) Plot of the average biconnectivity 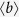 of the compounds of given closeness *c*. The correlation between *b* and *c* is *PCC* = 0.389. Inset: (left) Distribution of the closeness *c*. (right) Plot of 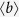 versus *c* with the log-binned data used. A crossover was identified from 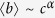 with 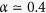 (solid line) to 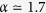 (dot-dashed line).

If a compound belongs to the LBC, it can maintain a connection to most core compounds; thus, its concentration can be readily stabilized, even when some pathways are inactive. We define the biconnectivity, *b*_*i*_, of a compound *i*, as the ratio of the number of species that have compound *i* in the LBC to the number of all species having it. The compound biconnectivity is expected to be related to the maintainability of the normal state against perturbations by utilizing alternative pathways. We demonstrated that among 6058 compounds 3071 compounds had *b* = 0, which indicates that they were *not* protected by backup pathways in their communication with the core compounds in *any* species. Many of these unprotected compounds were not isolated; they belong to the LCC. Defining the (single) connectivity *g*_*i*_ as the ratio of the number of species that have a compound *i* in the LCC to the number of all species that have it, we demonstrated that 5097 compounds had *g* = 1 and 2545 compounds had *g* = 1 and *b* = 0. We hypothesize that the abnormal concentration of a compound with low biconnectivity is not sufficiently lethal to the cells overall function as to overcome the entropic and biological costs of establishing backup pathways. In contrast, 971 compounds, approximately 16% overall, belonged to the LBC in *all* species. Chlorides, 3-beta-hydroxysterols, brassinosteroids, trans-polyisoprenyls, amino acids, quinones, folates, and uridine diphosphate glucose were the first few types of compounds that exhibited high biconnectivity. However, organosulfur, antibiotics, and sugar-alcohols exhibited quite low biconnectivity. The biconnectivity distribution *P*(*b*) peaked both at *b* = 0 and *b* = 1, whereas the connectivity distribution *P*(*g*) peaked at *g* = 1 [Fig. 4(a)].

We also evaluated the biconnectivity of each of the 924 pathways, as listed in the BioCyc database, via the mean biconnectivity of the involved compounds. When the analysis was restricted to the pathways consisting of more than 10 reactions, glycolysis had the largest biconnectivity *b* = 0.999, which indicates that the compounds involved in these pathways had that value of biconnectivity on average. Following the glycolysis pathways were L-histidine biosynthesis (*b* = 0.991), L-lysine biosynthesis (*b* = 0.989), adenosylcobalamin biosynthesis (*b* = 0.973), mixed acid fermentation (*b* = 0.962), and folate biosynthesis (*b* = 0.953). In contrast, nicotine degradation (*b* = 0.324) had the smallest biconnectivity, followed by the tRNA charging pathway (*b* = 0.382), the gibberellin inactivation pathway (*b* = 0.436), the phospholipase pathways (*b* = 0.524), and mycolate biosynthesis (*b* = 0.557).

Evolutionarily old compounds may have had more chances to acquire alternative pathways than young compounds over time, which effectively reduced the cost of establishing backup pathways. Relating the popularity *f* to the evolutionary age of each compound, one can expect a positive correlation between the popularity *f* and the biconnectivity *b*, which was confirmed by our analysis shown in Fig. 4(a). The correlation between the connectivity *g* and *f* was substantially weaker.

The metabolic compounds occupy different locations in each metabolic network and play different roles of varying importance in metabolism. On average, the compounds located close to other compounds may be lethal because their abnormality can quickly spread to other compounds. The deprivation of *essential* enzymes can halt the production of the biomass components, as well as cell growth^54^. For each compound *i*, we evaluated the average of the inverse of the network distance to the biomass components and denoted it by the closeness *c*_*i*_ to represent its potential lethality. We used the union of the biomass components identified for several well-studied species^55^, which may deviate from the true list of biomass components of each species. In ref. 55, the biomass components of *B. subtilis, E. coli, H. pylori, S. aureus, M. barkeri and S. cerevisiae* have been experimentally determined. Here, we consider the lethality of a compound as the probability that local perturbation around the compound can threaten the most important function of the metabolic network that generates the biomass components; furthermore, the compounds’ lethality may arise in a context other than their impact on biomass components. Figure 4(b) indicates that the biconnectivity *b* increases with the closeness *c*. This correlation implies that the metabolic networks have many loops around the biomass components, and the backup pathways are not equally distributed but concentrated around functionally important (lethal) compounds. Interestingly, we identified a crossover behavior *b* as a function of *c*: 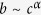, with 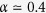 for small *c* and 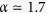 for large *c*, which indicates that the evolutionary pressure for backup pathways is substantially stronger for the compounds with high lethality.

### Implications of biconnectivity on the prevalence of human diseases

The malfunction of an enzyme accompanied by perturbation in the concentrations of related compounds is not confined and may spread in metabolism, which could result in a disorder on a large scale. Backup pathways help suppress these cascading failures. Human diseases, particularly metabolic diseases, may arise from cellular-level failures; thus, the prevalence pattern of various diseases may reflect the organization of backup pathways.

**Figure 5:**
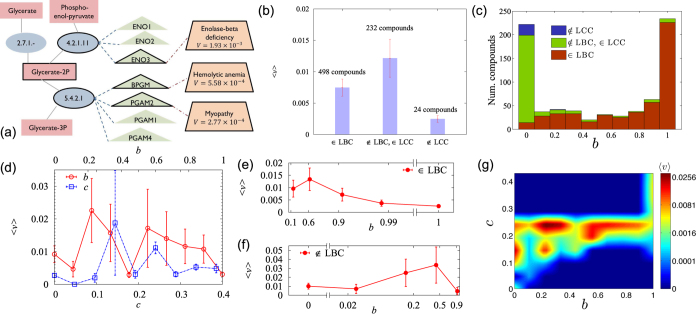
The vulnerability and the biconnectivity of the metabolic compounds. (**a**) The diseases associated with glycerate-2P. The compound (red square) is processed by three reactions (blue ellipses) denoted by the Enzyme Commission (EC) number. The genes (triangles) that generate the enzymatic proteins catalyzing the reactions are also shown, some of which are involved in the pathogenesis of three inherited diseases (trapezoids). The vulnerability of glycerate-2P is given by the mean of the prevalence values of the three diseases, 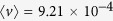. (**b**) Plots of the average vulnerability 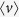 of the metabolic compounds in the LBC 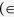 LBC), outside the LBC but in the LCC 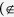 LBC, 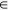 LCC), and outside the LCC 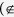 LCC) of the human metabolic network. (**c**) Distribution of the biconnectivity *b* for the three groups of the compounds considered in (**b**). (**d**) The average vulnerability 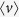 of the compounds of a given biconnectivity *b* and closeness *c*. The correlation between *v* and *b* was negative, 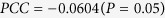. In contrast, the correlation between *v* and *c* was not significant, 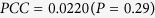. (**e**) Plot of 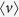 versus *b* for the compounds in the LBC. (**f**) Plot of 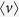 versus *b* for the compounds outside the LBC and in the LCC. (**g**) The vulnerability 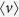 as a function of biconnectivity *b* and closeness *c*.

Inherited disorders are associated with the mutations of specific gene(s) and have been archived, e.g., in the Online Mendelian Inheritance in Man (OMIM) database^35^. In cases in which a disease gene generates enzymatic proteins that catalyze certain reactions, the perturbations of the compounds processed by the reactions may be involved in the pathogenesis of the associated diseases^24^,^37^. We consulted the OMIM database^35^ and the BioCyc database^8^ to identify the diseases potentially associated with each metabolic compound [Fig. 5(a)]. To assess the likelihood that a compound’s fluctuation would eventually lead to a human disease, we computed the mean prevalence of the associated disease. Here, the prevalence is the fraction of the patients diagnosed with a given disease among all patients in the Medicare dataset^24^,^25^,^36^,^37^. For each compound *i*, we took the mean prevalence of the associated diseases as its vulnerability *v*_*i*_.

The human metabolic network has 1513 compounds that are processed by 1451 reactions, and only 754 compounds are associated with 477 diseases. The compounds that have multiple pathways to the core compounds are less likely to cause diseases than the compounds without multiple pathways. In Fig. 5(b), the disease compounds in the LCC but outside the LBC of the human metabolic network have an average vulnerability 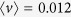, which is larger than 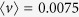 of the human LBC (*P* = 0.002). In contrast, the disease compounds located outside the LCC have 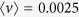, which is smaller than the compounds in the LCC because local fluctuations in the small components do not affect the LCC in which most elements reside.

An example of a disease that is associated with low-*b* and a high prevalence is depressive disorder with an accelerated response to antidepressant drug treatment, with 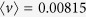. It is associated with peptidyl-proline, in which *b* = 0. Hyperproteinemia associated with angiotensinogen in which *b* = 0 has 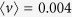. Renal carcinoma associated with 12 compounds that have 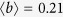 has 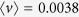. Other examples include renal tubular dysgenesis (*b* = 0, 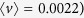 and dyskeratosis congenita (*b* = 0.0046 and 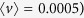.

Whether a disease compound belongs to the LBC and the LCC of the human metabolic network depends on the cross-species biconnectivity *b*. The disease compounds outside the human LBC have *b* = 0, with few exceptions [Fig. 5(c)]. Many disease compounds in the human LBC have *b* = 1, whereas some have *b* < 1. Diverse features of the human metabolic networks may contribute to preventing local perturbations from spreading, and the compound biconnectivity may provide the relevant information. The compounds with high biconnectivity are less vulnerable than the compounds that exhibit low biconnectivity. In Fig. 5(d), the vulnerability *v* is shown to decrease with the biconnectivity *b* 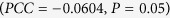. This correlation was stronger when we disregarded the currency metabolites in each metabolic network 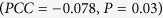 [Supplementary Material]. The vulnerability of the 498 compounds in the human LBC exhibited a negative correlation with their biconnectivity 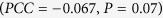, thus implying that the compounds with high biconnectivity are stabilized better than the compounds with low biconnectivity, while they are all in the human LBC.

The vulnerability of a compound depends on its lethality and its capacity to maintain its normal state, which can be roughly represented as follows:


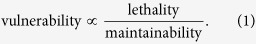


The closeness *c*_*i*_ of a compound can quantify its lethality, and the biconnectivity *b*_*i*_ represents a measure of the maintainability of the normal state under perturbations. The vulnerability *v* is expected to increase with the closeness *c* if other conditions are identical. However, the lethality and the maintainability are not independent of each other; evolutionary pressure is imposed to enhance the maintainability of lethal compounds, as supported by the correlation between the closeness *c* and the biconnectivity *b* shown in Fig. 4(b). The correlation between *b* and *c* makes it difficult to identify their independent contributions to vulnerability, as indicated in the weak correlation between *v* and *c* in Fig. 5(d). Interestingly, for compounds outside the human LBC, a higher biconnectivity is associated with greater vulnerability [Fig. 5(f)], which contrasts the negative correlation identified for the disease compounds in the human LBC. To understand the reason for this difference, let us assume that the lethality and the maintainability are proportional to the closeness *c* and the biconnectivity *b*, respectively, in Eq. (1). They are correlated with each other as shown in Fig. 4(b), in which 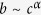 with 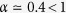 for small *c* 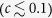 and 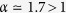 for large *c* 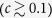. Inserting 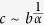 into 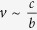 of Eq. (1), we find that 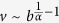. Therefore, *v* decreases with *b* if *α* > 1, which may be the case for the compounds in the human LBC that have a large *b*. However, *v* increases with *b* if *α* < 1, which is valid for the compounds outside the LBC that have a small *b*. Figure 5 (g) shows the plot of the vulnerability as a function of closeness *c* and biconnectivity *b*. Both plots in Fig. 5(e,f) indicate that *v* peaks at intermediate values of *b*, which results from *v* increasing with *b* for small *b* and decreasing with *b* for large *b*.

These findings suggest that the network properties of the metabolic compounds in other species can be helpful for understanding human diseases. The cross-species features reflect the generic features of each compound identified in diverse metabolic networks that have evolved in different ways; thus, they carry information that could not be identified by only analyzing the human metabolic network. Our findings could be useful in the search for drug targets and medical treatments. For example, if we must choose one of two candidate target compounds to set up alternative pathways and thereby suppress the spread of perturbations initiated somewhere in metabolism, we can first select the compound with increased biconnectivity. We expect this approach to establish the additional pathway and make it stable relatively easily.

## Discussion

Our study illustrates a novel use of the cross-species cellular network data that are rapidly accumulating in this post-genomic era. Metabolic pathway organization is essential to allow living organisms to be resilient to perturbations; thus, we performed an extensive quantitative analysis of the biconnectivity of the cellular metabolism in hundreds of species. As a result, we were able to understand how biconnectivity is achieved on small and large scales in real metabolic networks in the presence of other structural features, such as modularity and heterogeneous connectivity patterns. The idea that biconnectivity serves for resilience to perturbations is supported by the negative correlation between the prevalence of associated diseases and the biconnectivity of compounds. Furthermore, we demonstrated that the pattern of disease prevalence can be better understood by considering the dependence of the compound vulnerability on both the lethality and the biconnectivity, suggesting its potential use in drug design and medical treatments.

Our study does have several limitations. We used the biomass components known in the well-curated metabolic reconstructions^55^,^56^ as the biomass components of the species studied in our work as long as they were present in the metabolic network of a given species, which must be curated by plugging the species-specific biomass components. The development of a biconnectivity measure that incorporates the direction of chemical reactions could better elucidate pathway organization in metabolism. Although we focused on individual compounds, the properties of pairs of compounds in terms of their multiple paths deserve investigation. The present work focused on the structure of metabolic networks; however, an understanding of the dynamics, e.g., flux-balance modeling, would allow us to identify different sets of pathways that are active in wild-type and perturbation conditions, which would thereby facilitate an understanding of homeostatic mechanisms in living organisms. It would also be interesting to investigate structural biconnectivity and dynamical flux-coupling relations. The idea that biconnected compounds are resilient to perturbations can be confirmed and better supported by in-depth studies regarding the stoichiometry of specific compounds.

## Methods

### Database

We used the BioCyc database version 13.1^8^, which included 506 species, to construct the bipartite metabolic networks of 453 bacteria, 34 archaea, and 19 eukarya. The latest version is 19.1, which covers 5700 metabolic pathways as of 2015. The same compounds in different compartments were considered to reflect different nodes. The transport reactions between different compartments were included.

### Clustering coefficient of a bipartite network

A diamond-shaped subgraph (a quad) that involves two metabolic compounds and two reactions involves two disjoint pathways that connect the two compounds. For a bipartite metabolic network of the adjacency matrix *A*_*cr*_, we defined the clustering coefficient as follows:


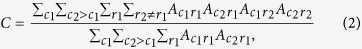


which is the ratio of the total number of quads that involve distinct pairs of compounds to the total number of reactions that involve pairs of compounds. If two compounds had *k* distinct reactions processing them, the number of quads would be *k*^2^. Therefore, *C* would be larger than 1 if all pairs of compounds had more than one reaction processing them. If all pairs of compounds were involved in only one reaction, *C* would be zero. This definition of the clustering coefficient is rather different from the previous definitions for bipartite networks^57^,^58^,^59^.

### Popularity, biconnectivity, and closeness

Let 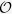 be the set of the 506 species considered in this work. Using 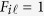 or 0 to represent whether a species 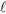 has a compound *i* or not, respectively, and 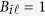 or 0 to represent whether a species 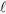 has the compound *i* in the LBC or not, respectively, we can represent the popularity and the biconnectivity as 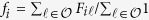 and 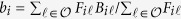, respectively. Considering the set 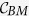 of the 129 biomass components identified in at least one species, i.e., *B. subtilis, E. coli, H. pylori, S. aureus, M. barkeri*, and *S. cerevisiae*^55^,^56^, we first made the set of biomass components 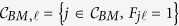 of a species 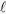. We subsequently computed the network distance 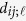 between a compound *i* and each biomass component 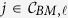 in each species 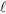 and evaluated the closeness as 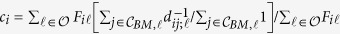.

## Additional Information

**How to cite this article**: Kim, P. *et al.* Biconnectivity of the cellular metabolism: A cross-species study and its implication for human diseases. *Sci. Rep.* **5**, 15567; doi: 10.1038/srep15567 (2015).

## Supplementary Material

Supplementary Information

## References

[b1] Horowitz N. H. On the evolution of biochemical syntheses. Proc. Natl. Acad. Sci. USA 31, 153 (1945). 16578152 10.1073/pnas.31.6.153 PMC1078786

[b2] Jensen R. A. Enzyme recruitment in evolution of new function. Annu. Rev. Microbiol. 30, 409 (1976). 791073 10.1146/annurev.mi.30.100176.002205

[b3] Yčas M. On earlier states of the biochemical system. J. theor. Biol. 44, 145 (1974). 4207200 10.1016/s0022-5193(74)80035-4

[b4] Light S. & Kraulis P. Network analysis of metabolic enzyme evolution in *Escherichia coli*. BMC Bioinformatics 5, 15 (2004). 15113413 10.1186/1471-2105-5-15 PMC394313

[b5] Yamada T. & Bork P. Evolution of biomolecular networks - lessons from metabolic and protein interactions. Nat. Rev. Mol. Cell Biol. 10, 791 (2009). 19851337 10.1038/nrm2787

[b6] Jeong H., Tombor B., Albert R., Oltvai Z. N. & Barabási A. L. The large-scale organization of metabolic networks. Nature 407, 651 (2000). 11034217 10.1038/35036627

[b7] Fell D. A. & Wagner A. The small world of metabolism. Nat. Biotech. 18, 1121 (2000). 10.1038/81025 11062388

[b8] Karp P. D. *et al.* Expansion of the BioCyc collection of pathway/genome databases to 160 genomes. Nucleic Acids Res. 33, 6083 (2005). 16246909 10.1093/nar/gki892 PMC1266070

[b9] Duarte N. C. *et al.* Global reconstruction of the human metabolic network based on genomic and bibliomic data. Proc. Natl. Acad. Sci. USA 104, 1777 (2007). 17267599 10.1073/pnas.0610772104 PMC1794290

[b10] Lee T. I. *et al.* Transcriptional Regulatory Networks in *Saccharomyces cerevisiae*. Science 298, 799 (2002). 12399584 10.1126/science.1075090

[b11] Babu M. M., Luscombe N. M., Aravind L., Gerstein M. & Teichmann S. A. Structure and evolution of transcriptional regulatory networks. Curr. Opin. Struct. Biol. 14, 283 (2004). 15193307 10.1016/j.sbi.2004.05.004

[b12] Uetz P. *et al.* A comprehensive analysis of protein-protein interactions in *Saccharomyces cerevisiae*. Nature 403, 623 (2000). 10688190 10.1038/35001009

[b13] Xenarios I. *et al.* DIP, the Database of Interacting Proteins: a research tool for studying cellular networks of protein interactions. Nucleic Acids Res. 30, 303 (2002). 11752321 10.1093/nar/30.1.303 PMC99070

[b14] Rual J.-F. *et al.* Towards a proteome-scale map of the human protein-protein interaction network. Nature 437, 1173 (2005). 16189514 10.1038/nature04209

[b15] Krogan N. J. *et al.* Global landscape of protein complexes in the yeast *Saccharomyces cerevisiae*. Nature 440, 637 (2006). 16554755 10.1038/nature04670

[b16] Barabási A.-L. & Oltvai Z. N. Network biology: understanding the cell’s functional organization. Nat. Rev. Genet. 5, 101 (2004). 14735121 10.1038/nrg1272

[b17] Albert R. Scale-free networks in cell biology. J. Cell Sci. 118, 4947 (2005). 16254242 10.1242/jcs.02714

[b18] Alon U. An Introduction to Systems Biology: Design Principles of Biological Circuits (Chapman and Hall/CRC, 2006).

[b19] Shen-Orr S. S., Milo R., Mangan S. & Alon U. Network motifs in the transcriptional regulation network of Escherichia coli. Nat. Genet. 31, 64 (2002). 11967538 10.1038/ng881

[b20] Ravasz E., Somera A., Mongru D., Oltvai Z. & Barabási A.-L. Hierarchical Organization of Modularity in Metabolic Networks. Science 297, 1551 (2002). 12202830 10.1126/science.1073374

[b21] Barabási A.-L. & Albert R. Emergence of Scaling in Random Networks. Science 286, 509 (1999). 10521342 10.1126/science.286.5439.509

[b22] Albert R. & Barabási A.-L. Statistical mechanics of complex networks. Rev. Mod. Phys. 74, 47 (2002).

[b23] Goh K.-I. *et al.* The human disease network. Proc. Natl. Acad. Sci. USA 104, 8685 (2007). 17502601 10.1073/pnas.0701361104 PMC1885563

[b24] Lee D.-S. *et al.* The implications of human metabolic network topology for disease comorbidity. Proc. Natl. Acad. Sci. USA 105, 9880 (2008). 18599447 10.1073/pnas.0802208105 PMC2481357

[b25] Park J., Lee D.-S., Christakis N. A. & Barabási A.-L. The impact of cellular networks on disease comorbidity. Mol. Sys. Biol. 5, 262(2009). 10.1038/msb.2009.16 PMC2683720 19357641

[b26] Barabasi A.-L., Gulbahce N. & Loscalzo J. Network medicine: a network-based approach to human disease. Nat. Rev. Genet. 12, 56 (2011). 21164525 10.1038/nrg2918 PMC3140052

[b27] Vidal M., Cusick M. E. & Barabási A.-L. Interactome Networks and Human Disease. Cell 144, 986 (2011). 21414488 10.1016/j.cell.2011.02.016 PMC3102045

[b28] Almaas E., Oltvai Z. N. & Barabási A.-L. The Activity Reaction Core and Plasticity of Metabolic Networks. PLoS Comput. Biol. 1, e68 (2005). 16362071 10.1371/journal.pcbi.0010068 PMC1314881

[b29] Albert R., Jeong H. & Barabási A.-L. Error and attack tolerance of complex networks. Nature 406, 378 (2000). 10935628 10.1038/35019019

[b30] Segre D., DeLuna A., Church G. M. & Kishony R. Modular epistasis in yeast metabolism. Nat. Genet. 37, 77 (2005). 15592468 10.1038/ng1489

[b31] Yeh P., Tschumi A. I. & Kishony R. Functional classification of drugs by properties of their pairwise interactions. Nat. Genet. 38, 489 (2006). 16550172 10.1038/ng1755

[b32] Ghim C.-M., Goh K.-I. & Kahng B. Lethality and synthetic lethality in the genome-wide metabolic network of *Escherichia coli*. J. theor. Biol. 237, 401 (2005). 15975601 10.1016/j.jtbi.2005.04.025

[b33] Shen Y. *et al.* Blueprint for antimicrobial hit discovery targeting metabolic networks. Proc. Natl. Acad. Sci. USA 107, 1082 (2010). 20080587 10.1073/pnas.0909181107 PMC2824290

[b34] Schmidt S., Sunyaev S., Bork P. & Dandekar T. Metabolites: a helping hand for pathway evolution? Trends Biochem. Sci. 28, 336 (2003). 12826406 10.1016/S0968-0004(03)00114-2

[b35] *Online mendelian inheritance in man, omim*®. McKusick-Nathans Institute of Genetic Medicine, Johns Hopkins Univer- sity (Baltimore, MD), World Wide Web URL: http://omim.org/ (Date of access : 21/02/2014).

[b36] Hidalgo C. A., Blumm N., Barabási A.-L. & Christakis N. A. A Dynamic Network Approach for the Study of Human Phenotypes. PLoS Comput. Biol. 5, e1000353 (2009). 19360091 10.1371/journal.pcbi.1000353 PMC2661364

[b37] Lee D.-S. Interconnectivity of human cellular metabolism and disease prevalence. J. Stat. Mech.: Theor. Exp. 2010, P12015 (2010).

[b38] Faust K., Dupont P., Callut J. & van Helden J. Pathway discovery in metabolic networks by subgraph extraction. Bioinformatics 26, 1211 (2010). 20228128 10.1093/bioinformatics/btq105 PMC2859126

[b39] Croes D., Couche F., Wodak S.J. & van Helden J. Metabolic PathFinding: inferring relevant pathways in biochemical networks. Nucleic Acids Res. 33, W326 (2005). 15980483 10.1093/nar/gki437 PMC1160198

[b40] Patil K. R. & Nielsen J. Uncovering transcriptional regulation of metabolism by using metabolic network topology. Proc. Natl. Acad. Sci. USA 102, 2685 (2005). 15710883 10.1073/pnas.0406811102 PMC549453

[b41] Hopcroft J. & Tarjan R. Algorithm 447: efficient algorithms for graph manipulation. Comm. ACM 16, 372 (1973).

[b42] Newman M. & Ghosal G. Bicomponents and the Robustness of Networks to Failure. Phys. Rev. Lett. 100, 138701 (2008). 18518004 10.1103/PhysRevLett.100.138701

[b43] Kim P., Lee D.-S. & Kahng B. Phase transition in the biconnectivity of scale-free networks. Phys. Rev. E 87, 022804 (2013). 10.1103/PhysRevE.87.022804 23496565

[b44] Lee D.-S., Goh K.-I., Kahng B. & Kim D. Evolution of scale-free random graphs: Potts model formulation. Nucl. Phys. B 696, 351 (2004).

[b45] Michal G. Biochemical Pathways: An Atlas of Biochemistry and Molecular Biology 2nd edn (eds Michal G. *et al.*) (Wi- ley, 1999).

[b46] Girvan M. & Newman M. E. J. Community structure in social and biological networks. Proc. Natl. Acad. Sci. USA 99, 7821 (2002). 12060727 10.1073/pnas.122653799 PMC122977

[b47] Clauset A., Newman M. E. J. & Moore C. Finding community structure in very large networks. Phys. Rev. E 70, 066111 (2004). 10.1103/PhysRevE.70.066111 15697438

[b48] Blondel V. D., Guillaume J.-L., Lambiotte R. & Lefebvre E. Fast unfolding of communities in large networks. J. Stat. Mech.: Theor. Exp. 2008, P10008 (2008).

[b49] Holme P. & Huss M. Currency metabolites and network representations of metabolism. arXiv:0806.2763v1 (2008).

[b50] Schellenberger J., Park J.O., Conrad T.M. & Palsson B. Ø. BiGG: a Biochemical Genetic and Genomic knowledgebase of large scale metabolic reconstructions. BMC Bioinformatics 11, 213 (2010). 20426874 10.1186/1471-2105-11-213 PMC2874806

[b51] Sayers E. W. *et al.* Database resources of the National Center for Biotechnology Information. Nucleic Acids Res. 37, D5 (2009). 18940862 10.1093/nar/gkn741 PMC2686545

[b52] Benson D. A., Karsch-Mizrachi I., Lipman D. J., Ostell J. & Sayers E. W. GenBank. Nucleic Acids Res. 37, D26 (2009). 18940867 10.1093/nar/gkn723 PMC2686462

[b53] Bernhardsson S., Gerlee P. & Lizana L. Structural correlations in bacterial metabolic networks. BMC Evol. Biol. 11, 20 (2011). 21251250 10.1186/1471-2148-11-20 PMC3033826

[b54] Palsson B. Ø. Systems Biology: Properties of Reconstructed Networks (Cambridge University Press, 2006).

[b55] *Systems biology research group: Downloads,* University of California (San Diego, CA), World Wide Web URL: http://systemsbiology.ucsd.edu/Downloads (Date of access : 31/12/2013).

[b56] Han K. Classification of organisms using backup reactions in metabolic networks. Master thesis, Seoul National University (2010).

[b57] Lind P. G., González M. C. & Herrmann H. J. Cycles and clustering in bipartite networks. Phys. Rev. E 72, 056127 (2005). 10.1103/PhysRevE.72.056127 16383708

[b58] Opsahl T. Triadic closure in two-mode networks: Redefining the global and local clustering coefficients. arXiv:1006.0887 (2010).

[b59] Kitsak M. & Krioukov D. Hidden variables in bipartite networks. Phys. Rev. E 84, 026114 (2011). 10.1103/PhysRevE.84.026114 21929071

[b60] Dietrich F. S. *et al.* The *Ashbya gossypii* Genome as a Tool for Mapping the Ancient Saccharomyces cerevisiae Genome. Science 304, 304 (2004). 15001715 10.1126/science.1095781

