# Supplementary material for: Biconnectivity of the cellular metabolism: A cross-species study and its implication for human diseases
Source: Sci Rep. 2015 Oct 22;5:15567. doi: 10.1038/srep15567 (PMC4614848; doi:10.1038/srep15567)
Supplement: Supplementary Information [file srep15567-s1.pdf]

Supplementary Material for "Biconnectivity of the cellular metabolism: A cross-species study and its implication for human diseases"

P. Kim,[1] D.-S. Lee,[2,*] and B. Kahng[1,†]

[1] *Center for Complex Systems Studies and CTP, Department of Physics and Astronomy, Seoul National University, Seoul 151-747, Korea*

[2] *Department of Physics, Inha University, Incheon 402-751, Korea*

I. BIPARTITE METABOLIC NETWORKS OF THE STUDIED SPECIES

Although the links of the constructed bipartite metabolic network can be directional for irreversible reactions, we made them undirected for simplicity and the possibility that variation in the reaction irreversibility depends on the environment: some irreversible reactions may be forced in the reverse direction under certain physiological conditions of temperature or metabolite concentrations [1–3]. The paths for each metabolic network are undirected, and therefore a path from a compound to another does not necessarily indicate the substrate - product relationship. For instance, a path of length 2 connecting two compounds c_1 and c_2 via a reaction r_1 may indicate that both are consumed (produced) together, or that one is converted to the other. We demonstrated that in the metabolism of a species, on average 1040 compounds were converted to one another by 960 reactions, with each reaction involving 4.5 compounds. The mean degree $\bar{k} = 2L/N$ of the real metabolic networks was not small, ranging between 3 and 5.

* deoksun.lee@inha.ac.kr

† bkahng@snu.ac.kr

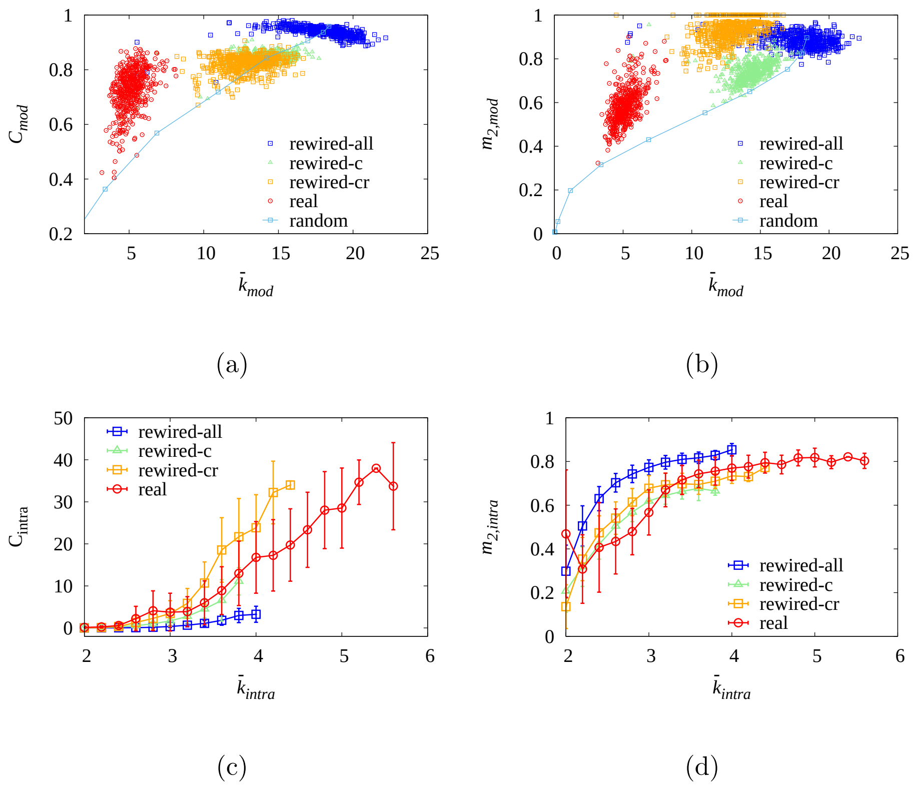

FIG. S1. Modular and intra-module biconnectivity. (a) The clustering coefficient of the modular networks, $C_{\rm mod}$, as a function of the modular mean degree $\bar{k}_{\rm mod}$, evaluated following the conventional definition, $C_{\rm mod} = \sum_{i,j,k} A_{ij}A_{ik}A_{jk} / \sum_{i,j,k} A_{ij}A_{ik}$ with A_{ij} the adjacency matrix of the modular network. The line indicates the clustering coefficient of the modular networks extracted from the random ER networks. (b) The fraction of the LBC, $m_{2,\rm mod}$, in the modular networks. (c) The plots of the clustering coefficient of the modules themselves, $C_{\rm intra}$, versus the intra-module mean degree $\bar{k}_{\rm intra} = 2L_{\rm intra}/N_{\rm intra}$, where $N_{\rm intra}$ and $L_{\rm intra}$ are the number of nodes and links within each module. The data are binned. (d) The fraction of the LBC within modules, $m_{2,\rm intra}$, as a function of $\bar{k}_{\rm intra}$.

II. IDENTIFYING MODULES: MODULAR AND INTRA-MODULE BICONNECTIVITY

To identify the modules of each metabolic network, we used two methods, which gave the same results qualitatively. To determine the community each node i belongs to by maximizing the modularity Q, which represents to what degree there are intra-community

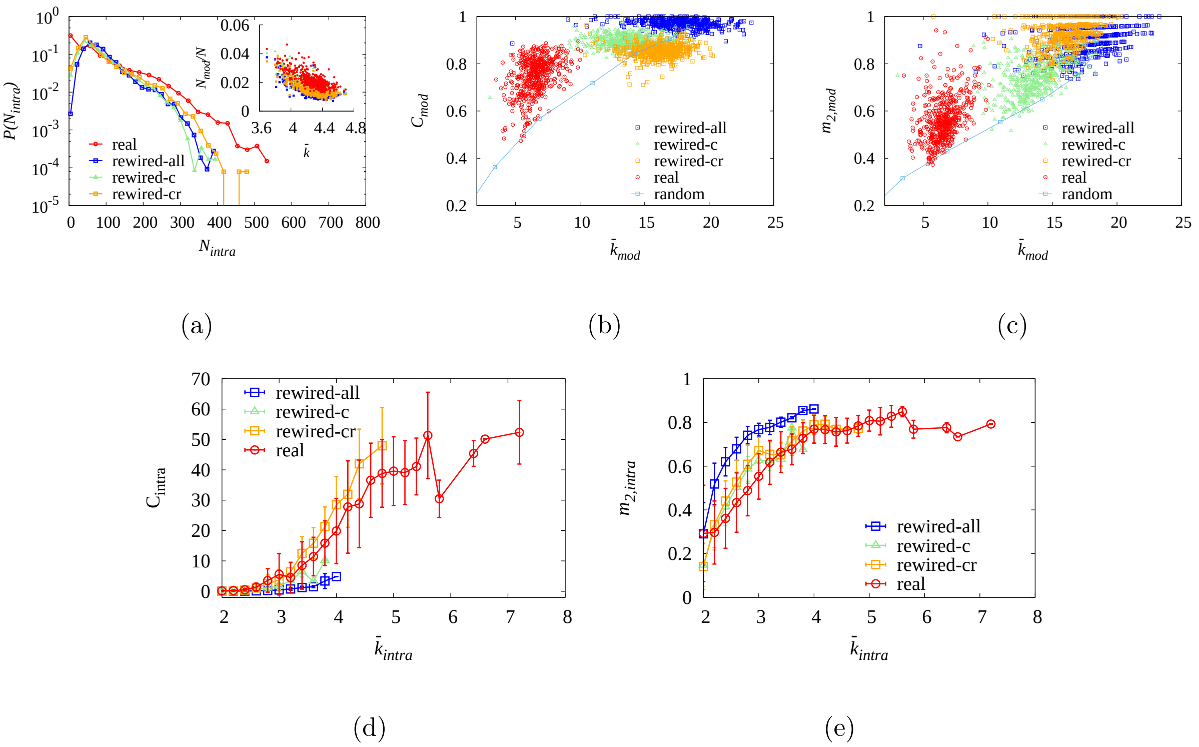

FIG. S2. Modular and inter-module biconnectivity investigated by using the algorithm in Ref. [5]. (a) Distribution of the size of a module $N_{\rm intra}$. Inset: Plot of the number of modules $N_{\rm mod}$ versus $\bar{k} = 2L/N$. (b) The clustering coefficient of the modular networks, $C_{\rm mod}$, as a function of the modular mean degree $\bar{k}_{\rm mod}$. (c) The fraction of the LBC, $m_{2,{\rm mod}}$, in the modular networks. (d) The plots of the clustering coefficient of the modules themselves, $C_{\rm intra}$, versus the intra-module mean degree $\bar{k}_{\rm intra} = 2L_{\rm intra}/N_{\rm intra}$. (e) The fraction of the LBC within modules, $m_{2,{\rm intra}}$, as a function of $\bar{k}_{\rm intra}$.

links compared with random networks, and is defined as [4]

$$Q = \frac{1}{2L}\sum_{i=1}^{N}\sum_{j=1}^{N}\left[A_{ij} - \frac{k_i k_j}{2L}\right]\delta_{c_i,c_j}, \tag{S1}$$

where $k_i = \sum_{j=1}^{N} A_{ij}$, $L = (1/2)\sum_{i=1}^{N} k_i$.

Connecting the modules if a link connects the nodes that belong to them in the original network, one can obtain the modular network of $N_{\rm mod}$ nodes and $L_{\rm mod}$ links. The biconnectivity of those modular networks extracted from the real and the rewired networks for each species is shown in Fig. S1 (a) and (b). The mean degree $\bar{k}_{\rm mod} = 2L_{\rm mod}/N_{\rm mod}$ is larger in the rewired-all and rewired-c networks than in the real and the rewired-cr networks. For comparison, we present $C_{\rm mod}$ and $m_{2,{\rm mod}}$ of the modular networks extracted from the ER

networks, which we called the random modular networks. The biconnectivity of the real and rewired-cr networks has larger values of C_{mod} and $m_{2,\text{mod}}$ than the random modular networks for a given k_{mod}. However, the rewired-all and the rewired-c networks have values of C_{mod} and $m_{2,\text{mod}}$ that are similar to the random modular networks for a given k_{mod}.

The biconnectivity of the nodes *within* each module is also shown in [Fig. S1 (c) and (d)]. Clustering coefficient of the modules themselves, C_{intra}, is greater in the rewired-cr networks than the real networks while clustering coefficient, C, of the whole network is greater in the real networks than the rewired-cr networks (Fig. 3 (a) in the main text). The fraction of the LBC within modules shows no significant differences between the real and rewired-cr networks.

To confirm whether our results depend on the method of identifying modules, we used the method in Ref. [5] to extract the modules. This algorithm uncovers a complete hierarchical partitions, providing different resolutions of community structure for the network. It can be seen that the results given in Fig. S2 are not different from those obtained using the first method qualitatively.

III. METABOLIC NETWORKS WITH CURRENCY METABOLITES REMOVED

Some metabolites, called currency metabolites [6], are highly abundant, and fluctuation in their concentration has little impact on other metabolites. Therefore, it was necessary to determine whether our results were changed when those currency metabolites were removed from the metabolic networks. It should be noted that because currency metabolites are generally abundant, their removal can lead to a reduction in the connectivity of the whole metabolic network.

We used the algorithm proposed in Ref. [6] to identify the currency metabolites in each species' metabolic network. Protons, water, ATP, ADP, phosphate, polyphosphate, carbon dioxide, AMP, NADPH, NADP and oxygen were classified as the currency metabolites in at least one species. The biconnectivity of the metabolic networks *with the currency metabolites and their links removed* are given in Fig. S3, which confirms that our results remain valid even if the currency metabolites are ignored.

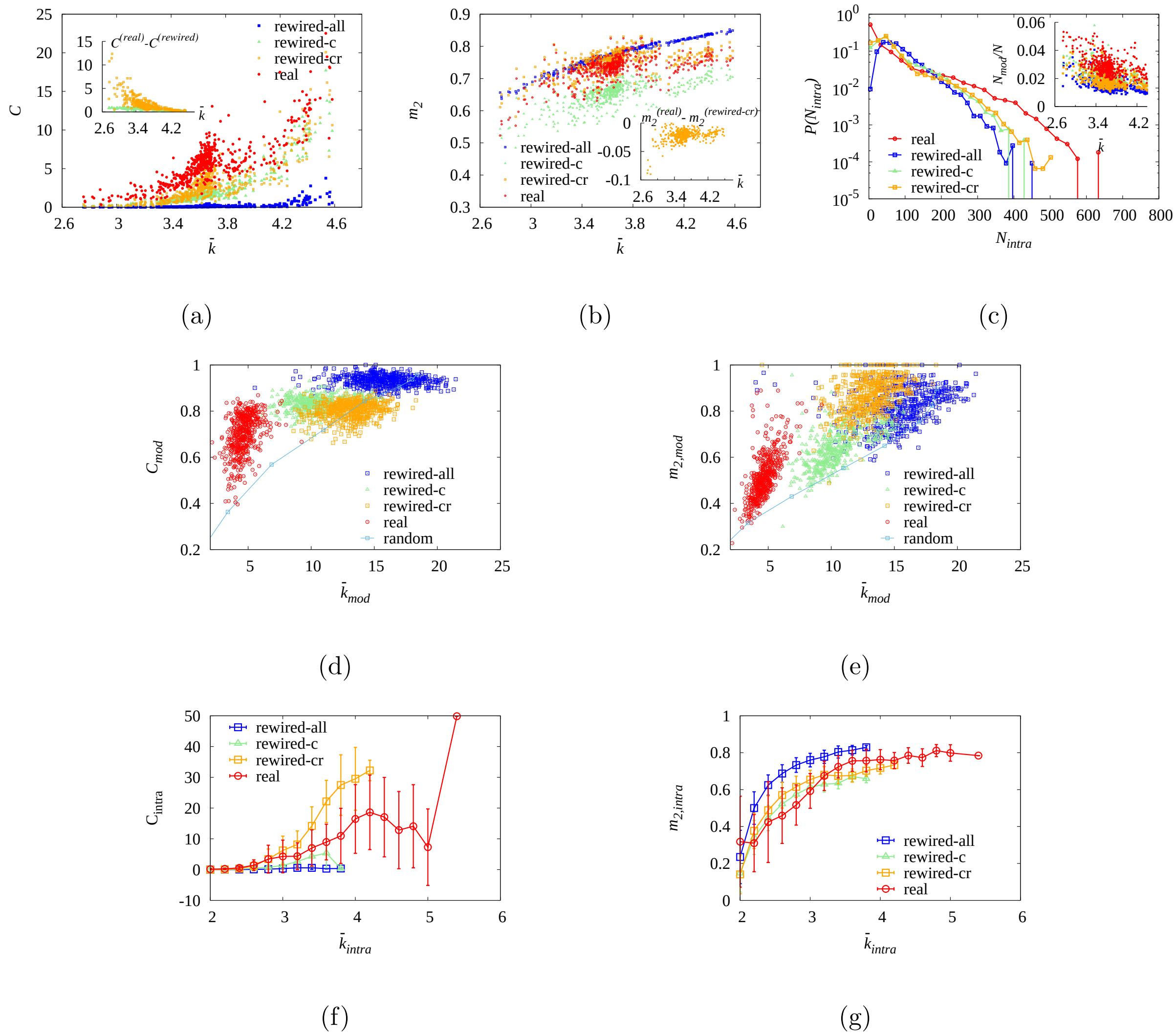

FIG. S3. Biconnectivity of the metabolic networks without the currency metabolites. (a) Plot of the clustering coefficient C versus the mean degree $\bar{k}$. (b) Plot of the fraction of the LBC m_2 versus the mean degree $\bar{k}$. (c) Distribution of the size of a module $N_{\rm intra}$. Inset: Plot of the number of modules $N_{\rm mod}$ versus $\bar{k}$. (d) The modular clustering coefficient $C_{\rm mod}$ versus the mean degree of the modular networks $\bar{k}_{\rm mod}$. (e) The fraction of the modular LBC, $m_{2,\rm mod}$, versus $\bar{k}_{\rm mod}$. (f) The plots of the clustering coefficient of the modules themselves, $C_{\rm intra}$, versus the intra-module mean degree $\bar{k}_{\rm intra} = 2L_{\rm intra}/N_{\rm intra}$. (g) The fraction of the LBC within modules, $m_{2,\rm intra}$, as a function of $\bar{k}_{\rm intra}$.

IV. METABOLIC NETWORKS OF NINE SPECIES FROM THE BIGG DATABASE

To confirm the robustness of our results, we investigated the well-curated metabolic reconstructions of nine strains; They are two strains of *Escherichia coli*, *Helicobacter pylori, Pseudomonas putida, Staphylococcus aureus, Methanosarcina barkeri, Mycobacterium tuberculosis, Saccharomyces cerevisiae*, and *Homo sapiens*, which are available in the BiGG database [7]. The analysis of these metabolic networks further confirms our main conclusions.

V. EVOLUTION OF BICONNECTIVITY

We defined the normalized differences of the clustering coefficients and the fractions of the LBC between the two species i and j as follows:

$$\delta C_{ij} = \frac{|C_i - C_j|}{C_i + C_j}, \quad \delta m_{2,ij} = \frac{|m_{2,i} - m_{2,j}|}{m_{2,i} + m_{2,j}} \tag{S2}$$

and computed them with the real metabolic networks and the rewired networks. The normalized differences increased with the phylogenetic distance, d_{ij}, because the evolutionarily-close species pairs tend to have similar number of nodes and links in their metabolism. To remove such trivial similarity, we considered the ratio of the normalized difference between the rewired networks to that between the real networks for each pair of species: $\left(\frac{\delta C_{ij}^{(\mathrm{real})}}{\delta C_{ij}^{(\mathrm{rewired-all})}}\right)^{\nu}$, $\left(\frac{\delta C_{ij}^{(\mathrm{real})}}{\delta C_{ij}^{(\mathrm{rewired-c})}}\right)^{\nu}$, $\left(\frac{\delta m_{2,ij}^{(\mathrm{real})}}{\delta m_{2,ij}^{(\mathrm{rewired-all})}}\right)^{\nu}$, and $\left(\frac{\delta m_{2,ij}^{(\mathrm{real})}}{\delta m_{2,ij}^{(\mathrm{rewired-c})}}\right)^{\nu}$, where an exponent ν is introduced. We demonstrated that with $\nu \lesssim 0.6$, the ratios of δC and δm_2 to the values in the rewired-all networks exhibited positive and significant correlation with the phylogenetic distance, d_{ij} [Fig. S5]. When the values of ν were smaller than 1, the contributions of small values were significant compared to the average. However, a significant correlation was not observed for the ratio to the rewired-c networks.

VI. POPULARITY OF THE METABOLIC COMPOUNDS

The popularity of metabolic compounds is broadly distributed as shown in Fig. S6 (a). Although we took the popularity of a compound as its evolutionary age, the evolutionary

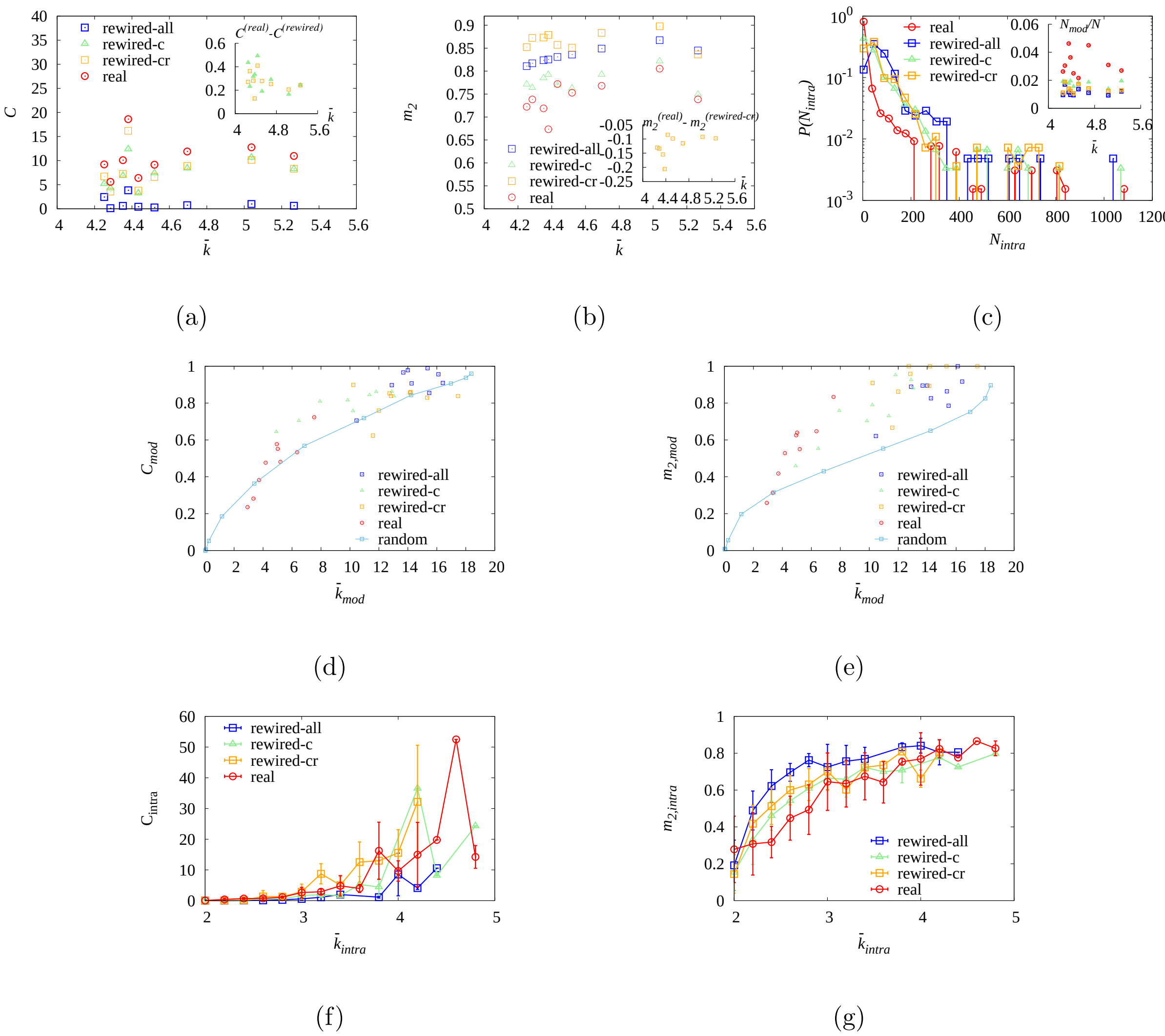

FIG. S4. Biconnectivity of the well-curated metabolic networks of two strains of *E. coli*, *H. pylori, P. putida, S. aureus, M. barkeri, M. tuberculosis, Saccharomyces cerevisiae*, and *Homo sapiens* from the BiGG database. (a) Plot of the clustering coefficient, C, versus the mean degree $\bar{k}$. (b) Plot of the fraction of the LBC, m_2, versus the mean degree $\bar{k}$. (c) Distribution of the size of a module N_{intra}. Inset: Plot of the number of modules N_{mod} versus $\bar{k}$. (d) The modular clustering coefficient C_{mod} versus the mean degree of the modular networks $\bar{k}_{\text{mod}}$. (e) The fraction of the modular LBC, $m_{2,\text{mod}}$, versus $\bar{k}_{\text{mod}}$. (f) The plots of the clustering coefficient of the modules themselves, C_{intra}, versus the intra-module mean degree $\bar{k}_{\text{intra}} = 2L_{\text{intra}}/N_{\text{intra}}$. (g) The fraction of the LBC within modules, $m_{2,\text{intra}}$, as a function of $\bar{k}_{\text{intra}}$.

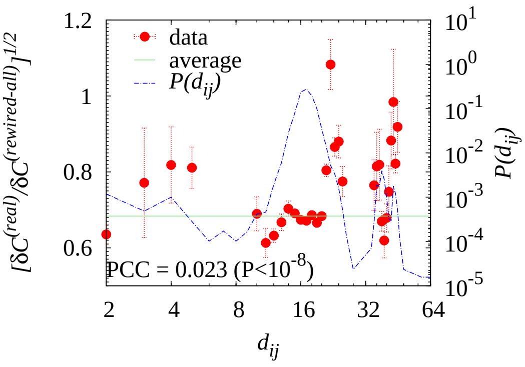

(a)

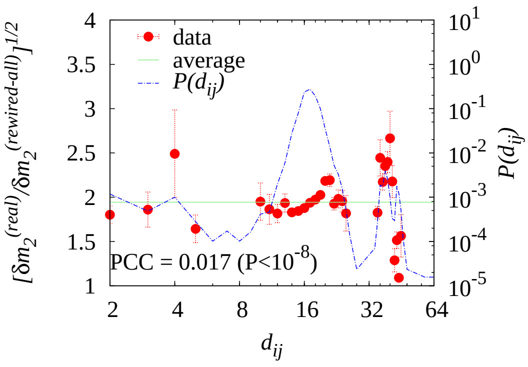

(b)

FIG. S5. Similarity of biconnectivity between two species. (a) Plot of the square-root of the ratio of the normalized difference of the clustering coefficients of two species i and j in their real networks to that in their 'rewired-all' networks, $\left(\frac{\delta C^{(\mathrm{real})}_{(i,j)}}{\delta C^{(\mathrm{rewired-all})}_{(i,j)}}\right)^{1/2}$, versus the evolutionary distance d_{ij}. (b) Plot of the square-root of the ratio of the normalized difference of the m_2 of two species i and j in their real networks to that in their 'rewired-all' networks, $\left(\frac{\delta m^{(\mathrm{real})}_{2,(i,j)}}{\delta m^{(\mathrm{rewired-all})}_{2,(i,j)}}\right)^{1/2}$, versus their distance d_{ij}.

age can be better estimated by performing parsimony analysis [8, 9] with the phylogenetic trees of the studied set of species [10, 11]. We demonstrated that the popularity was also negatively correlated with the vulnerability (P-value 0.04): the older a compound was, the lower its vulnerability. Old compounds have played core roles in metabolism for a long time, therefore the metabolic network may have been remodeled during evolution to prevent them from becoming abnormal.

Although popularity and biconnectivity were positively correlated [Fig. 3 (b)], they seem to have made independent contributions to the vulnerability [Fig. S6 (b)]. For the 417 young compounds with $f < 0.5$, the less biconnected ($b < 0.5$) compounds had higher vulnerability ($\langle v \rangle = 0.011$) than the more biconnected ($b \geq 0.5$) ones. The 337 old compounds with $f \geq 0.5$ showed a similar behavior: the vulnerability was higher for the less biconnected compounds ($\langle v \rangle = 0.085$) than the more biconnected ones ($\langle v \rangle = 0.0062$).

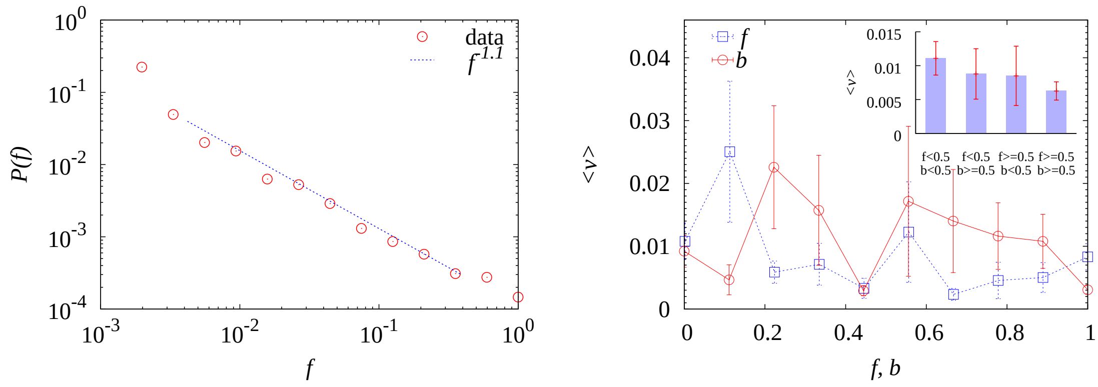

(a) (b)

FIG. S6. (a)Distribution of the compound popularity f. The line represents $P(f) \sim f^{-1.1}$, which fits the data points. (b) Plots of the vulnerability versus the biconnectivity b and the popularity f. Inset: The vulnerability of the four groups of compounds classified according to their popularity and biconnectivity.

VII. CROSS-SPECIES BICONNECTIVITY AND CLOSENESS WITH THE CURRENCY METABOLITES DISREGARDED

To confirm the robustness of our results, we considered the biconnectivity and the closeness of each compound obtained from the metabolic networks where the currency metabolites were removed. The biconnectivity, b_i, of a compound i was defined as the ratio of the number of species that have the compound i in the LBC of the metabolic network *with the currency metabolites and their links removed*, to the number of all the species that have the compound. Also, the closeness c_i was the average of the inverse of the network distance to the known biomass components in the metabolic networks *with the currency metabolites and their links removed*. The same analyses as in Figs. 4 and 5 of the main text, but obtained by using the cross-species biconnectivity and closeness with the currency metabolites disregarded are presented in Figs. S7 and S8. This confirms the robustness of our results which investigate the cross-species properties of metabolites in the main text.

VIII. STUDIED SPECIES

Our study was on the following 506 species, with different strains of a species considered distinct:

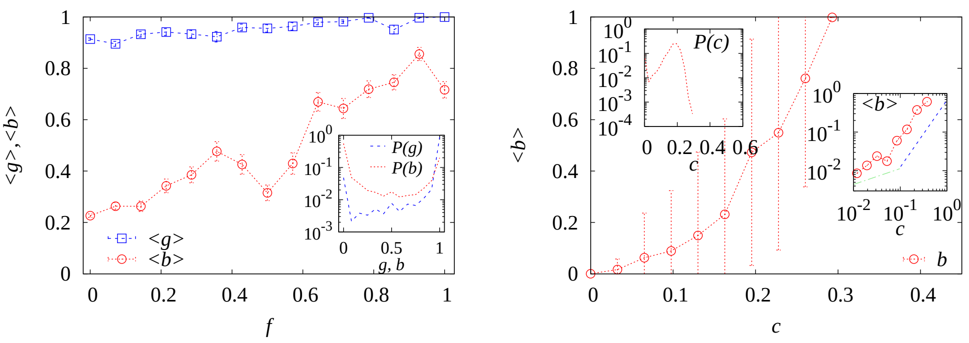

(a) (b)

FIG. S7. The cross-species biconnectivity of the metabolic compounds, the same analyses as in Fig. 3 of the main text, with the currency metabolites disregarded in the metabolic networks of each species for computing the biconnectivity b and the closeness c of each compound. (a) The average (single-) connectivity $\langle g\rangle$ and the biconnectivity $\langle b\rangle$ of the compounds of given popularity f. The correlation between the g and f was $PCC = 0.101$ and that between b and f was $PCC = 0.371$. Inset: The distributions of g and b. (b) Plot of the average biconnectivity $\langle b\rangle$ of the compounds of given closeness c. The correlation between b and c was $PCC = 0.415$. Inset: (left) Distribution of the closeness c. (right) Plot of $\langle b\rangle$ versus c with the log-binned data used. A crossover can be seen from $\langle b\rangle \sim c^{\alpha}$ with $\alpha \simeq 0.4$ (solid line) to $\alpha \simeq 1.7$ (dot-dashed line).

A. aeolicus VF5, A. avenae citrulli AAC00-1, A. bacterium Ellin345, A. borkumensis SK2, A. cellulolyticus 11B, A. cryptum JF-5, A. dehalogenans 2CP-C, A. ehrlichei MLHE-1, A. ferrooxidans ATCC 23270, A. fulgidus DSM 4304, A. gossypii ATCC 10895, A. tumefaciens C58, A. hydrophila dhakensis, A. marginale St. Maries, A. metalliredigens QYMF, A. naeslundii MG1, B. anthracis Ames, A. pernix K1, A. phagocytophilum HZ, A. pleuropneumoniae L20, A. salmonicida salmonicida A449, Arthrobacter sp., A. sp. JS42, A. sp. BH72, A. sp. ADP1, A. aromaticum EbN1, A. tumefaciens C58, A. sp. SI85-9A1, A. variabilis ATCC 29413, B. abortus bv. 1 str., B. afzelii PKo, B. ambifaria AMMD, B. ambifaria MC40-6, B. anthracis Sterne, B. anthracis Ames Ancestor, B. aphidicola Sg (Schizaphis graminum), B. aphidicola Bp (Baizongia pistaciae), B. aphidicola Cc (Cinara cedri), B. avium 197N, B. bacteriovorus HD100, B. bacilliformis KC583, B. bronchiseptica RB50, B. burgdorferi B31, B. cenocepacia HI2424, B. cereus ATCC 14579, B. cereus E33L, B. cereus cytotoxis NVH 391-98, B. clausii KSM-K16, B. fragilis NCTC 9343, B. fragilis YCH46, B. garinii PBi, B. halodurans C-125, B. henselae Houston-1, B. japon-

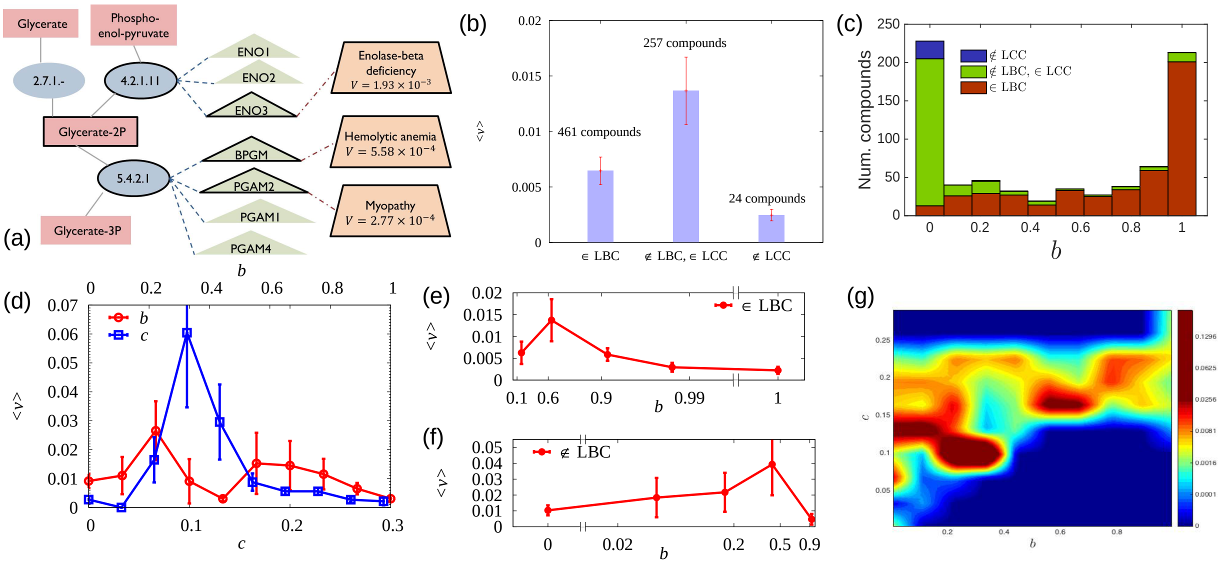

FIG. S8. The vulnerability and biconnectivity of the metabolic compounds with the biconnectivity and closeness evaluated by considering the metabolic networks with the currency metabolites and their links removed. (a) The same figure as Fig. 4 (a). (b) Plots of the average vulnerability $\langle v \rangle$ of the metabolic compounds in the LBC ($\in$ LBC), outside the LBC but in the LCC ($\notin$ LBC, $\in$ LCC), and outside the LCC ($\notin$ LCC) of the human metabolic network. (c) Distribution of the biconnectivity b for the three groups of compounds considered in (b). (d) The average vulnerability $\langle v \rangle$ of the compounds of a given biconnectivity b and closeness c. The correlation between v and b was negative, $PCC = -0.0781\,(P = 0.03)$ and that between v and c was $PCC = -0.121\,(P = 0.001)$. (e) Plot of $\langle v \rangle$ versus b for the compounds in the LBC. (f) Plot of $\langle v \rangle$ versus b for the compounds outside the LBC and in the LCC. (g) The vulnerability $\langle v \rangle$ as a function of biconnectivity b and closeness c.

icum USDA 110, B. licheniformis ATCC 14580, B. licheniformis ATCC 14580, B. longum NCC2705, B. mallei ATCC 23344, B. mallei SAVP1, B. melitensis 16M, B. melitensis biovar Abortus 2308, B. ovis, B. parapertussis 12822, B. pertussis Tohama I, B. pseudomallei K96243, B. pseudomallei 1710b, B. pumilus SAFR-032, B. quintana Toulouse, B. aphidicola APS (Acyrthosiphon pisum), Burkholderia sp., Bradyrhizobium sp., B. suis 1330, B. thailandensis E264, B. thetaiotaomicron VPI-5482, B. thuringiensis serovar konkukian str., B. thuringiensis Al Hakam, B. turicatae 91E135, B. aphidicola, B. vietnamiensis G4, B. weihenstephanensis KBAB4, B. xenovorans LB400, C. abortus S26/3, C. acetobutylicum ATCC 824, B. taurus, C. crescentus CB15, C. beijerinckii NCIMB 8052, C. Blochman-

nia floridanus, C. Blochmannia pennsylvanicus BPEN, C. botulinum A, C. botulinum A str. ATCC, C. botulinum A str. Hall, C. botulinum F str. Langeland, C. botulinum B1 str. Okra, C. botulinum A3 str. Loch, C. burnetii RSA 493, C. burnetii RSA 331, C. burnetii Dugway 5J108-111, C. Carsonella ruddii PV, C. caviae GPIC, C. chlorochromatii CaD3, C. difficile 630, C. diphtheriae NCTC 13129, C. efficiens YS-314, C. felis Fe/C-56, C. glutamicum ATCC 13032, C. glutamicum ATCC 13032, C. hutchinsonii ATCC 33406, C. hydrogenoformans Z-2901, C. jeikeium K411, C. jejuni jejuni NCTC 11168, C. jejuni RM1221, C. kluyveri DSM 555, C. Methanoregula boonei 6A8, C. michiganensis michiganensis NCPPB 382, C. muridarum Nigg, C. novyi NT, C. parvum Iowa, C. Pelagibacter ubique HTCC1062, C. perfringens 13, C. perfringens ATCC 13124, C. perfringens SM101, C. phytofermentans ISDg, C. pneumoniae AR39, C. pneumoniae CWL029, C. pneumoniae J138, C. pneumoniae TW-183, C. Protochlamydia amoebophila UWE25, C. psychrerythraea 34H, C. Ruthia magnifica Cm (Calyptogena magnifica), C. salexigens DSM 3043, C. tepidum TLS, C. tetani E88, C. trachomatis A/HAR-13, C. trachomatis A/HAR-13, C. Vesicomyosocius okutanii HA, C. violaceum ATCC 12472, D. aromatica RCB, D. desulfuricans desulfuricans G20, D. ethenogenes 195, D. geothermalis DSM 11300, D. hafniense Y51, Drosophila melanogaster, D. psychrophila LSv54, D. radiodurans R1, D. sp. BAV1, D. sp. CBDB1, D. vulgaris Hildenborough, E. canis Jake, P. atrosepticum SCRI1043, E. chaffeensis Arkansas, E. coli K-12 substr. MG1655, E. coli O111:H-, E. coli CFT073, E. coli K-12 substr. W3110, E. coli 536, E. coli UTI89, E. coli APEC O1, E. coli SMS-3-5, E. coli O157:H7, E. coli O157:H7 EDL933, Encephalitozoon cuniculi, E. litoralis HTCC2594, E. ruminantium Welgevonden, E. ruminantium Welgevonden, E. ruminantium Gardel, Enterobacter sp., F. alni ACN14a, F. johnsoniae UW101, F. nucleatum nucleatum ATCC 25586, F. tularensis tularensis SCHU S4, F. succinogenes succinogenes S85, F. tularensis holarctica FSC200, F. tularensis holarctica OSU18, F. tularensis tularensis FSC198, F. novicida U112, F. tularensis tularensis WY96-3418, G. bethesdensis CGDNIH1, G. kaustophilus HTA426, G. metallireducens GS-15, G. oxydans 621H, G. sulfurreducens PCA, G. thermodenitrificans NG80-2, G. violaceus PCC 7421, H. acinonychis Sheeba, H. utahensis DSM 12940, H. arsenicoxydans, H. butylicus DSM 5456, H. chejuensis KCTC 2396, H. ducreyi 35000HP, H. halophila SL1, H. hepaticus ATCC 51449, H. influenzae 86-028NP, H. influenzae Rd KW20, H. marismortui ATCC 43049, H. neptunium, C. hominis TU502, H. pylori 26695, H. pylori HPAG1, H. pylori J99, H. somnus 129PT, H. sp. NRC-1, H. sapiens, H. walsbyi DSM 16790, I. loihiensis L2TR, J.

sp. CCS1, J. sp. Marseille, K. pneumoniae pneumoniae MGH 78578, L. acidophilus NCFM, L. brevis ATCC 367, L. casei ATCC 334, L. delbrueckii bulgaricus ATCC BAA-365, L. delbrueckii bulgaricus ATCC 11842, L. innocua Clip11262, Leptospira interrogans, L1-130, L. interrogans serovar Lai str., L. intracellularis PHE/MN1-00, L. johnsonii NCC 533, L. lactis lactis Il1403, L. mesenteroides mesenteroides ATCC 8293, L. monocytogenes EGD-e, L. monocytogenes 4b F2365, L. plantarum WCFS1, L. pneumophila pneumophila Philadelphia 1, L. pneumophila Lens, L. pneumophila Paris, L. pneumophila Corby, L. reuteri DSM 20016, L. sakei sakei 23K, L. welshimeri serovar 6b str., L. xyli xyli CTCB07, M. acetivorans C2A, M. aquaeolei VT8, M. arthritidis 158L3-1, M. avium 104, M. barkeri Fusaro, M. bovis AF2122/97, M. bovis BCG str. Pasteur, M. burtonii DSM 6242, M. capsulatus Bath, M. capricolum capricolum ATCC 27343, M. flagellatus KT, M. florum L1, M. genitalium G37, M. gilvum PYR-GCK, M. hyopneumoniae J, M. hyopneumoniae 7448, M. hyopneumoniae 232, M. kandleri AV19, M. labreanum Z, M. leprae TN, M. loti MAFF303099, M. magneticum AMB-1, M. maripaludis S2, M. maris MCS10, M. maripaludis C5, M. maripaludis C7, M. mazei Go1, M. mobile 163K, M. mycoides mycoides SC str., M. musculus, M. penetrans HF-2, M. petroleiphilum PM1, M. pneumoniae M129, M. pulmonis UAB CTIP, M. smegmatis MC2 155, M. sp. MCS, M. sp. KMS, M. sp. MWYL1, M. stadtmanae DSM 3091, M. synoviae 53, M. tuberculosis CDC1551, M. tuberculosis H37Rv, M. thermautotrophicus Delta H, M. thermoacetica ATCC 39073, M. thermophila PT, M. tuberculosis F11, M. tuberculosis H37Ra, M. vanbaalenii PYR-1, M. xanthus DK 1622, Neurospora crassa, N. europaea ATCC 19718, N. farcinica IFM 10152, N. gonorrhoeae FA 1090, N. hamburgensis X14, N. meningitidis MC58, N. meningitidis Z2491, N. meningitidis FAM18, N. multiformis ATCC 25196, N. sennetsu Miyayama, N. sp. PCC 7120, Nocardioides sp., N. winogradskyi Nb-255, O. carboxidovorans OM5, O. iheyensis HTE831, O. tsutsugamushi Boryong, P. abyssi GE5, P. acnes KPA171202, P. aerophilum IM2, P. aeruginosa UCBPP-PA14, P. aeruginosa PAO1, P. arcticus 273-4, O. yellows phytoplasma, P. atlantica T6c, P. berghei ANKA, P. carbinolicus DSM 2380, P. chabaudi, P., P. cryohalolentis K5, P. distasonis ATCC 8503, P. entomophila L48, P. fluorescens Pf0-1, P. fluorescens Pf-5, P. furiosus DSM 3638, P. gingivalis W83, P. haloplanktis TAC125, P. horikoshii OT3, P. intermedia 17, P. islandicum DSM 4184, P. falciparum 3D7, P. luminescens laumondii TTO1, P. luteolum DSM 273, P. marinus AS9601, P. marinus marinus CCMP1375, P. marinus pastoris MED4ax, P. marinus NATL1A, P. marinus NATL2A, P. marinus MIT 9312, P.

marinus MIT 9313, P. mendocina ymp, P. naphthalenivorans CJ2, P. pentosaceus ATCC 25745, P. profundum SS9, P. putida F1, P. ruminicola 23, Pirellula sp., P. sp. JS666, P. necessarius asymbioticus QLW-P1DMWA-1, P. stutzeri A1501, P. syringae pv. syringae B728a, P. syringae pv. tomato str., P. thermopropionicum SI, P. torridus DSM 9790, P. vivax SaI-1, P. yoelii yoelii 17XNL, R. bellii RML369-C, R. conorii Malish 7, R. denitrificans OCh 114, R. etli CFN 42, R. eutropha JMP134, R. eutropha H16, R. felis URRWX-Cal2, R. ferrireducens T118, R. leguminosarum bv. viciae 3841, R. metallidurans CH34, R. metallidurans CH34, R. palustris CGA009, R. palustris BisA53, R. palustris BisB18, R. palustris BisB5, S. pomeroyi DSS-3, R. prowazekii Madrid E, R. rubrum ATCC 11170, R. salmoninarum ATCC 33209, R. solanacearum GMI1000, R. jostii RHA1, R. sphaeroides 2.4.1, R. sphaeroides ATCC 17029, R. sphaeroides ATCC 17025, R. typhi Wilmington, R. xylanophilus DSM 9941, S. acidocaldarius DSM 639, S. aciditrophicus SB, S. agalactiae A909, S. agalactiae NEM316, S. alaskensis RB2256, S. aureus aureus Mu50, S. aureus aureus N315, S. aureus aureus MW2, S. aureus RF122, S. aureus aureus MRSA252, S. aureus aureus MSSA476, S. aureus aureus JH9, S. aureus aureus JH1, S. aureus aureus USA300, S. aureus aureus Newman, S. aureus aureus NCTC 8325, S. aureus aureus COL, S. avermitilis MA-4680, S. boydii Sb227, S. coelicolor A3(2), S. degradans 2-40, S. dysenteriae Sd197, S. elongatus PCC 7942, S. elongatus PCC 6301, S. enterica enterica serovar Typhi, S. enterica enterica serovar Typhi, S. enterica enterica serovar Paratyphi, S. enterica enterica serovar Choleraesuis, S. epidermidis RP62A, S. epidermidis ATCC 12228, S. erythraea NRRL 2338, S. flexneri 2a str. 301, S. flexneri 5 str. 8401, S. glossinidius morsitans, S. gordonii Challis, S. haemolyticus JCSC1435, S. flexneri 2a str. 2457T, S. loihica PV-4, S. mansoni, S. marinus F1, S. medicae WSM419, S. meliloti 1021, S. mutans UA159, S. oneidensis MR-1, S. pneumoniae R6, S. pneumoniae Hungary19A-6, Schizosaccharomyces pombe, S. pyogenes M1 GAS, S. pyogenes MGAS8232, S. pyogenes SSI-1, S. pyogenes MGAS315, S. pyogenes MGAS10394, S. pyogenes MGAS5005, S. pyogenes MGAS6180, S. pyogenes MGAS9429, S. pyogenes MGAS10270, S. pyogenes MGAS2096, S. pyogenes MGAS10750, S. ruber DSM 13855, S. saprophyticus saprophyticus ATCC 15305, S. solfataricus P2, S. sonnei Ss046, Synechococcus sp., S. sp. PCC 6803, S. sp. TM1040, S. sp. WH 7803, Shewanella sp., S. sp. CC9311, S. sp. WH 8102, S. sp. ANA-3, S. sp. CC9311, S. suis 05ZYH33, S. suis 98HAH33, S. thermophilus LMG 18311, S. thermophilum IAM 14863, S. thermophilus CNRZ1066, S. thermophilus LMD-9, S. tokodaii 7, S. tropica CNB-440, S. typhimurium

LT2, B. subtilis subtilis 168, S. wolfei wolfei Goettingen, T. acidophilum DSM 1728, T. crunogena XCL-2, T. denticola ATCC 35405, T. denitrificans ATCC 25259, S. denitrificans DSM 1251, T. elongatus BP-1, T. erythraeum IMS101, T. fusca YX, T. kodakarensis KOD1, T. maritima MSB8, T. gondii ME49, T. pallidum pallidum Nichols, T. petrophila RKU-1, T. brucei, T. tengcongensis MB4, T. thermophilus HB27, T. thermophilus HB8, T. volcanium GSS1, T. whipplei Twist, T. whipplei TW08/27, u. methanogenic archaeon RC-I, U. parvum serovar 3 str., U. urealyticum serovar 13, V. cholerae O1 biovar El, V. eiseniae EF01-2, V. fischeri ES114, V. parahaemolyticus RIMD 2210633, V. vulnificus YJ016, V. vulnificus CMCP6, W. endosymbiont of Brugia malayi, W. pipientis, W. succinogenes DSM 1740, X. axonopodis pv. citri str., X. campestris pv. campestris str., X. campestris pv. campestris str., X. campestris pv. vesicatoria str., X. fastidiosa 9a5c, X. fastidiosa Temecula1, X. oryzae pv. oryzae KACC10331, X. oryzae pv. oryzae MAFF, X. oryzae pv. oryzae PXO99A, S. cerevisiae S288c, Y. enterocolitica enterocolitica 8081, Y. pestis KIM, Y. pestis CO92, Y. pestis biovar Microtus str., Y. pestis Angola, Y. pestis Antiqua, Y. pestis Nepal516, Y. pestis Pestoides F, Y. pseudotuberculosis IP 32953, Z. mobilis mobilis ZM4

IX. BIOMASS COMPONENTS

To compute the closeness of each compound, we considered the set of the biomass components directly measured in at least one of the well-curated metabolic reconstructions of *B. subtilis, E. coli, H. pylori, S. aureus, M. barkeri and S. cerevisiae* by Dr. Palssons research group [12]. In Ref. [12], the biomass components of *B. subtilis, E. coli, H. pylori, S. aureus, M. barkeri and S. cerevisiae* have been experimentally determined. We assume that every species shares these biomass components, which may deviate from the true list of biomass components.

As a result, we demonstrated 129 biomass components as follows:

1,2-diacylglycerol, 1,3-beta-d-glucan, carbon dioxide, 10-formyl-tetrahydrofolate, 2-octaprenyl-6-hydroxyphenol, 3'-phosphoadenylyl-sulfate, 5,10-methylenetetrahydrofolate, tetrahydrofolate, 5-methyl-tetrahydrofolate, AMP, ATP, acetyl-coA, citrate lyase apo-[acyl-carrier-protein], citrate lyase holo-[acyl-carrier protein], malonate-decarboxylase-apo-[acyl-carrier-protein], malonate-decarboxylase-holo-[acyl-carrier-protein], water, CTP, adenosylcobalamin, adenosylcobalamin-5'-phosphate, ammonia, ammonium-hydroxide, biotin, CDP,

CMP, cardiolipin, chloride, coA, coa-disulfide, coa-glutathione, cu2+, d-glucosamine-1-phosphate, 3-d-glucosyl-1,2-diacylglycerol, ergosterol, fe2+, fe3+, FAD, FADH2, GDP, GMP, GTP, glycine, heme-o, kdo2-lipid-iva, L-alanine, L-arginine, L-asparagine, L-aspartate, L-cysteine, L-glutamate, L-glutamine, L-histidine, L-isoleucine, L-leucine, L-lysine, L-methionine, L-phenylalanine, L-proline, L-serine, L-threonine, L-tryptophan, L-tyrosine, L-valine, ll-diaminopimelate, meso-diaminopimelate, lipopolysaccharide, glucomannan, phosphomannan, menaquinone-6, mn2+, molybdate, n-acetyl-glucosamine-1-phosphate, n-acetyl-d-glucosamine, NAD, NAD+, NADH, NADP, NADP+, NADPH, peptidoglycan, phosphatidat, 1,2-diacyl-sn-glycerol-3-phosphate, phosphatidylcholine, l-1-phosphatidyl-ethanolamine, l-1-phosphatidyl-glycerol, l-1-phosphatidylserine, protoheme, protoheme-ix, putrescine, pyridoxal-5'-phosphate, pyrophosphate, reduced-riboflavin, riboflavin, s-adenosyl-l-methionine, siroheme, spermidine, succinyl-coA, sulfate, thiamine, thiamine-diphosphate, trehalose, udp-n-acetylmuramate, udp-d-glucose, UMP, UTP, undecaprenyl-diphosphate, coenzyme-f390-a, coenzyme-f430, coenzyme-b, coenzyme-alpha-f420-3, reduced-coenzyme-gamma-f420-2, coenzyme-M, dAMP, dATP, dCMP, dCTP, dGMP, dGTP, dTMP, dTTP, glycogen, mRNA, 1-phosphatidyl-d-myo-inositol, l-1-phosphatidyl-inositol, tetrahydrosarcinapterin, triacylglycerol, zymosterol.

---

[1] Faust, K., Dupont, P., Callut, J. & van Helden, J. Pathway discovery in metabolic networks by subgraph extraction. *Bioinformatics* **26**, 1211 (2010).

[2] Croes, D., Couche, F., Wodak, S.J. & van Helden, J. Metabolic PathFinding: inferring relevant pathways in biochemical networks. *Nucleic Acids Res.* **33**, W326 (2005).

[3] Patil, K.R. & Nielsen, J. Uncovering transcriptional regulation of metabolism by using metabolic network topology. *Proc. Natl. Acad. Sci. USA* **102**, 2685 (2005).

[4] Clauset, A., Newman, M. E. J. & Moore, C. Finding community structure in very large networks. *Phys. Rev. E* **70**, 066111 (2004).

[5] Blondel, V.D., Guillaume, J.-L., Lambiotte, R. & Lefebvre, E. Fast unfolding of communities in large networks. *J. Stat. Mech.: Theor. Exp.* **2008**, P10008 (2008).

[6] Holme, P. & Huss, M. Currency metabolites and network representations of metabolism. arXiv:0806.2763v1 (2008).

[7] Schellenberger, J., Park, J.O., Conrad, T.M. & Palsson, B.Ø. BiGG: a Biochemical Genetic and Genomic knowledgebase of large scale metabolic reconstructions. *BMC Bioinformatics* **11**, 213 (2010).

[8] Campillos, M., von Mering, C., Jensen, L. J. & Bork, P. Identification and analysis of evolutionarily cohesive functional modules in protein networks. *Genome Res.* **16**, 374 (2006).

[9] Borenstein, E., Kupiec, M., Feldman, M. W. & Ruppin, E. Large-scale reconstruction and phylogenetic analysis of metabolic environments. *Proc. Natl. Acad. Sci. USA* **105**, 14482 (2008).

[10] Bernhardsson, S., Gerlee, P. & Lizana, L. Structural correlations in bacterial metabolic networks. *BMC Evol. Biol.* **11**, 20 (2011).

[11] Caetano-Anollés, G., Kim, H. S. & Mittenthal, J. E. The origin of modern metabolic networks inferred from phylogenomic analysis of protein architecture. *Proc. Natl. Acad. Sci. USA* **104**, 9358 (2007).

[12] *Systems biology research group: Downloads, University of California (San Diego, CA), World Wide Web URL: http://systemsbiology.ucsd.edu/Downloads (Date of access : 31/12/2013).*
